# Recent progress in probing small molecule interactions with DNA

**DOI:** 10.1007/s12551-025-01373-z

**Published:** 2025-11-27

**Authors:** Simon Poole, Bríonna McGorman, Christine J. Cardin, Andrew Kellett

**Affiliations:** 1 School of Chemical Sciences, Dublin City University, Glasnevin, Dublin 9, Ireland; 2 Department of Chemistry, University of Reading, Whiteknights, Reading, RG6 6AD UK

**Keywords:** DNA, Nucleic acids, B-DNA, Non-canonical DNA, Triple helix, Triplex-forming oligonucleotides, Three-way junction, Holliday junction, Drug-DNA interactions

## Abstract

Nucleic acids are primary therapeutic targets, and understanding drug-DNA interactions is essential to the discovery of new clinical agents. In recent years, the desire to develop therapies with specific biological targets has produced new molecules that preferentially interact with complex nucleic acid sequences and structures. As such, the targeting of non-canonical nucleic acids, including DNA triplexes, G-quadruplexes, i-motifs, three-way junctions and Holliday junctions, have emerged due to their roles in gene regulation, genome stability and cellular stress responses. Characterising the interactions of these non-canonical structures with new ligands and metal complexes has led to the discovery of promising agents with therapeutic potential. Biophysical techniques including spectroscopic methods, crystallography and biomolecular assays have been critical to probing these interactions. This review describes recent advancements in the analysis of higher-order drug-DNA interactions for the rational design of targeted therapeutics.

## Introduction

**Fig. 1:**
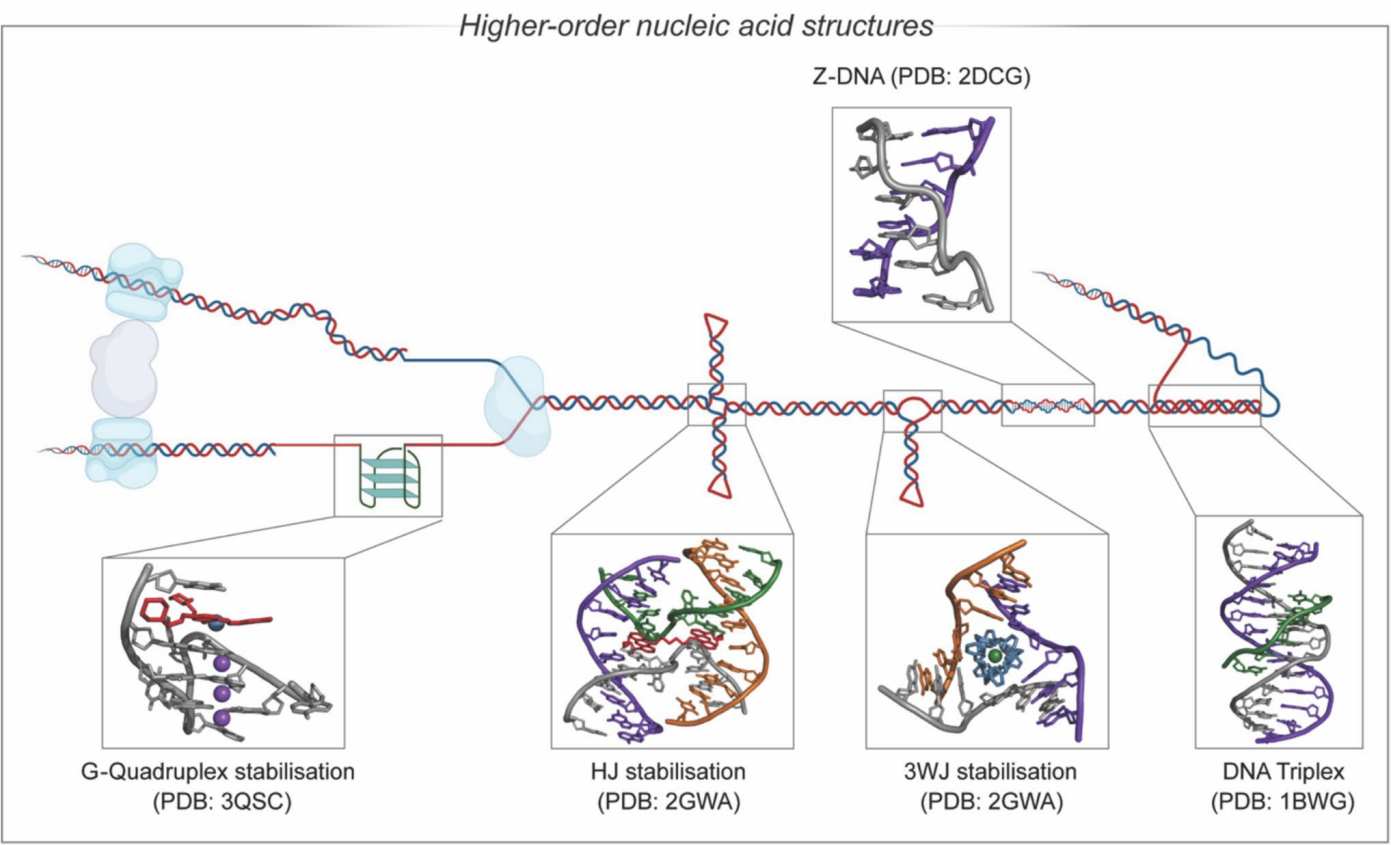
Higher-order non-canonical nucleic acid structures including G-quadruplex, HJ, 3WJ, Z-DNA and DNA triplex structures. (Figure adapted from Zell et al. 2020*.*)

The structure of B-DNA was first reported in 1953 by Watson and Crick, Franklin and Wilkins (Watson and Crick 1953; Franklin and Gosling 1953; Wilkins et al. 1953). Since then, variations of this nucleic acid (NA) structure have been described, including A-DNA and Z-DNA (Dickerson et al. 1982; Wing et al. 1980), which arise in specific physiological conditions. Higher-order structures that do not adhere to canonical Watson–Crick base pairing have also been reported, such as DNA triplexes, three-way junctions (3WJ), four-way junctions including Holliday junctions (HJs), G-quadruplexes (Prieto Otoya et al. 2024b) and i-motifs (Fig. 1). These non-canonical nucleic acids are structurally distinct and play critical roles in biological processes, and consequently are of significant interest as therapeutic targets (Tateishi-Karimata and Sugimoto 2020). Analysis of these DNA structures is of importance as it enables an evaluation of how drug molecules interact with canonical and non-canonical DNA thereby providing avenues for new targeted therapeutics.

This review firstly introduces duplex B-DNA, and its structural analogues, A- and Z-DNA, before describing DNA triplexes and the therapeutic potential of triplex-forming oligonucleotides (TFOs), followed thereafter by junction DNA—including 3WJs and the HJ—where techniques employed for the biophysical analysis are described using specific compounds that can interact with these higher-order NAs (Table 1).

**Table 1:** Examples of agents used to target dsDNA, 3WJs and HJs

Target	Compound	Key non-covalent interactions
dsDNA	Intercalators	π stacking between successive base pairs
dsDNA	Groove binders	Electrostatic and H-bonding within the major or minor DNA grooves
dsDNA	TFOs	Sequence-selective binding at Hoogsteen face (major groove)
3WJ	Iron(II) helicates	Hydrophobic interactions, cavity fitting
3WJ	Copper(II) peptide helicates	Recognition via hydrophobic interactions within the cavity; covalently modifies structure upon oxidative damage via ROS generation
3WJ	Triptycene derivatives	Stabilisation via hydrophobic and π-stacking interactions in junction core
HJ	Bis-Acridines (C6)	Stabilisation via base displacement and intercalation between CG base pairs; forms pseudo-base pairs with thymine bases
HJ	Organometallic pillarplexes	Hydrophobic binding to the open-X central cavity; induces cavity contraction
HJ	VE-822	Potentially promotes assembly and stabilisation of HJs; induces DNA damage response

### Duplex DNA 

**Fig. 2:**
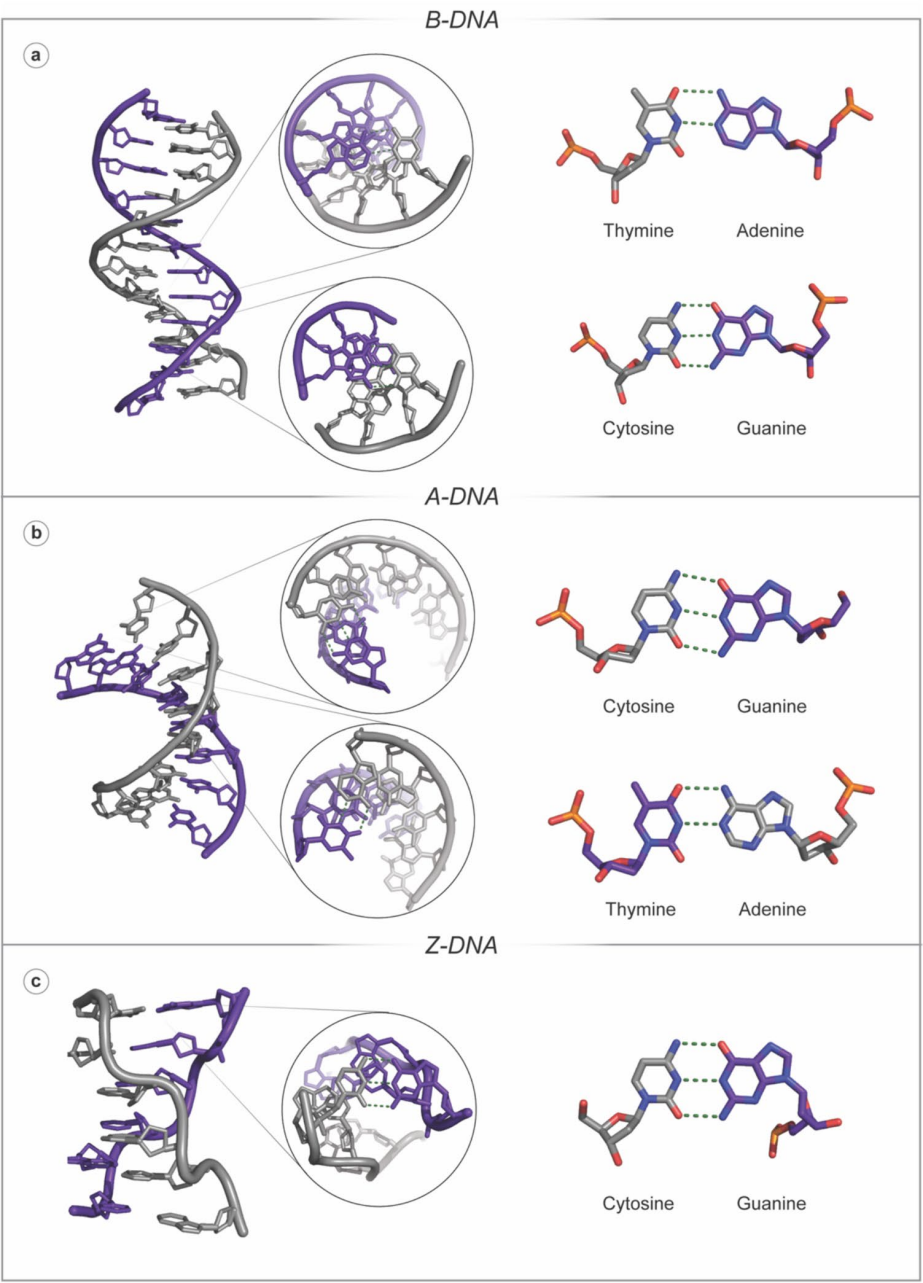
**a** B-DNA (PDB: 1BNA) indicating a top-down and side view of Watson–Crick base-pairing between T:A and C:G nucleobases, showing the *anti* glycosyl angle conformation, the C2ʹ-endo sugar pucker conformation and the right-handed helical sense. (Purine bases A and G are purple, and pyrimidine bases T and C are grey); **b** A-DNA (PDB: 1VJ4) indicating a top-down and side view of Watson–Crick base-pairing between T:A and C:G nucleobases, showing the *anti*-glycosyl angle conformation, the C3ʹ-endo sugar pucker conformation and the right-handed helical sense; **c** Z-DNA (PDB: 2DCG) indicating a top-down and side view of Watson–Crick base-pairing between C:G nucleobases, showing the *anti* glycosyl angle conformation for cytosine and the *syn* conformation for guanine, the C2ʹ-endo sugar pucker conformation for cytosine and the C3'-endo sugar pucker conformation for guanine and the left-handed helical sense

DNA is composed of two deoxyribose NA strands that run in an anti-parallel orientation (Watson and Crick 1953). The backbone is composed of repeating negatively charged sugar-phosphate groups that link successive heteroaromatic purine nucleobases, adenine (A) and guanine (G), and pyrimidine nucleobases, cytosine (C) and thymine (T). Both chains in the DNA duplex are held together by non-covalent nucleobase interactions that obey Chargaff’s rule whereby a 1:1 ratio of purine:pyrimidine bases interact via hydrogen bonding (Fig. 2) (Chargaff 1971). A notable structural consequence of A:T and C:G base pairing is the generation of unequal major and minor grooves in B-DNA (Kellett et al. 2019b).

 Duplex DNA morphology constitutes B-DNA, A-DNA and Z-DNA (Fig. 2), which feature a major and minor groove. These grooves arise due to asymmetry in the sugar-phosphate backbone, primarily from the antiparallel orientation of the two DNA strands and the nature of the deoxyribose sugar unit in the backbone (Watson and Crick 1953). This furanose ring features an exocyclic C5′ carbon, which extends outside the ring, connecting the phosphate group through an oxygen atom. The resultant structure creates uneven spacing between the backbone chains, as the glycosidic bonds that tether the DNA bases to C1′ of the sugar are angled and thus offset relative to the base pairs, creating a difference in spacing in the DNA backbone, known as the major and minor grooves. Each duplex form—B-, A- and Z-DNA—has distinct parameters such as glycosidic torsion angle (*χ*), sugar pucker conformation and helical sense (Dickerson et al. 1982). Specific structures arise depending on the physiological environment, and the three forms can generally be easily distinguished (Kellett et al. 2019b) using circular dichroism (CD) spectroscopy (Ghoshdastidar and Senapati 2018; Miyahara et al. 2013). B-DNA is the most common form encountered under normal physiological conditions. It has a right-handed helical sense with 10 residues per turn, generating a specific axial rise and helical diameter. B-DNA follows canonical Watson–Crick base pairing and has exceptional stability due to π-π stacking interactions between successive heteroaromatic nucleobases within the helix. A-DNA is a dehydrated form of B-DNA and, as such, has the same right-handed helical sense as B-DNA. Interestingly, A-DNA has been investigated as an evolutionary defence mechanism in desiccant-resistant prokaryotic species, whereby dehydrated specimens undergo conformation change from B- to A-DNA *en masse* and maintain biological function post-rehydration (Whelan et al. 2014). Finally, Z-DNA has a notably different structure to B- and A-DNA as it presents a zig-zag sugar-phosphate backbone and has a left-handed helical sense. This form of DNA requires an environment with a high salt concentration, which stabilises the proximity of the phosphate backbone between nucleobases in this conformation. It can maintain this zig-zag backbone formation as it is primarily composed of alternating purine(Pu):pyrimidine(Py)—d(PuPy) or d(PyPu)—nucleobases with *syn:anti* conformations, respectively, although sequences of d(GGGC)_n_—which do not follow the alternating purine:pyrimidine motif—have been shown to adopt a Z-DNA conformation (Wang and Vasquez 2007). Recent discoveries have highlighted the significance of the conformational change to Z-DNA for memory formation in critical biological processes, with regulatory roles in the prefrontal cortex of mice for a learned lessening of fear (fear extinction) (Marshall et al. 2020). The ability of B-DNA to flip to the Z-conformation under conditions of superhelical stress, coupled with the existence of Zα and Zβ binding domains (found in enzymes such as Adenosine Deaminase Acting on RNA 1 (ADAR1), and Z-DNA Binding Protein 1 (ZBP1) which bind Z-DNA, respectively) suggests that the conversion to Z-DNA is important in biological processes (Wang and Vasquez 2007; Marshall et al. 2020).

### Advancements in dsDNA targeted therapies

Duplex DNA has been the main structural target and focus for therapeutic drug development for many years, with various classes of drugs developed to exploit its structural and functional vulnerabilities. Classic DNA-damaging agents include activated bleomycin (Chen and Stubbe 2005), platinum drugs (Farrell 2015; Johnstone et al. 2016), doxorubicin (Anders et al. 2013) and cyclophosphamide (Colvin 1999). Multiple examples of successful anti-cancer drugs that specifically interact with DNA have been reviewed elsewhere (Kellett et al. 2019b; Chen and Stubbe 2005; Gibney and Kellett 2024; Kelland 2007), including prominent examples of platinum drugs, bleomycin, topoisomerase I inhibitors, netropsin and distamycin-based analogues.

**Fig. 3:**
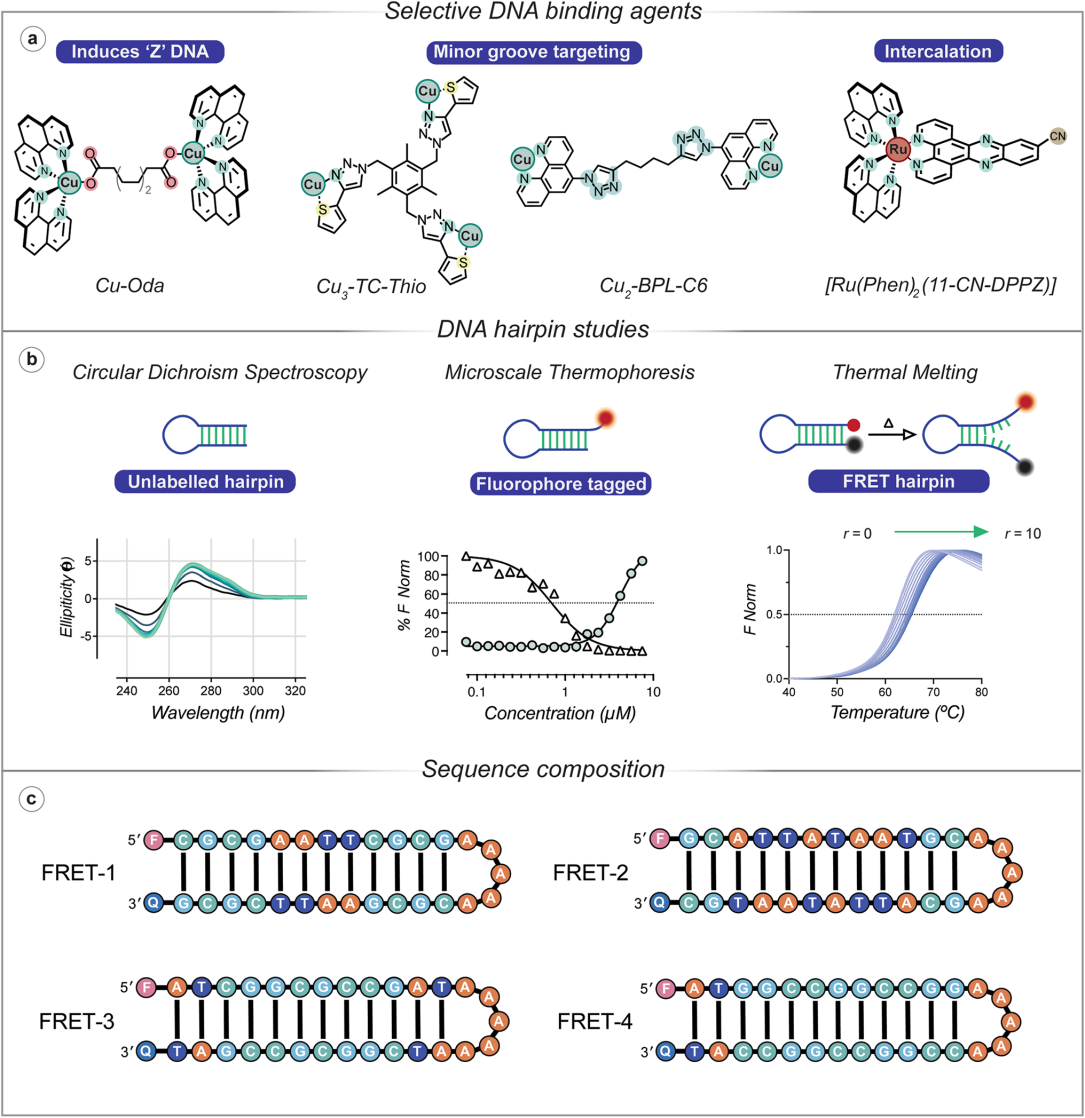
**a** Chemical structures of DNA binding agents: Cu-Oda (Oda = octanedioate), which induces ‘Z-like’ DNA formation; Cu_2_-BPL-C6 (BPL-C6 = bis-1,10-Phenanthroline-carbon-6) and Cu_3_-TC-Thio (TC-Thio = tri-click thiophene), both targeting the minor groove of DNA; and [Ru(Phen)_2_(11-CN-DPPZ)]^2+^ (Phen = 1,10 = Phenanthroline, and DPPZ = dipyridophenazine), which intercalates DNA; **b** analytical techniques demonstrating binding efficacy: *Circular Dichroism Spectroscopy* showing ellipticity (mdeg) versus wavelength (nm) for an unlabelled DNA hairpin, reflecting structural changes upon compound binding; *Microscale Thermophoresis* displaying normalised fluorescence (% F Norm) versus concentration (µM) for a 5′-fluorophore-tagged hairpin, quantifying binding affinity; *Thermal Melting* with a 5′-fluorophore and 3′-quencher illustrating normalised fluorescence (*F*_Norm_) versus temperature (°C) with *r* = [drug]/[DNA] ranging from 0 to 10, indicating the thermal stability of metal complex bound DNA-; **c** schematic of FRET DNA hairpins (5′-fluorophore and 3′-quencher) with varied AT and GC content, which enable sequence specific binding interactions to be evaluated

Copper-based metallodrugs offer different mechanisms of action to clinically approved therapeutics, which may overcome resistance associated with traditional treatments. Artificial metallo-nucleases (AMNs) have been at the forefront due to their ability to generate oxidative DNA lesions via reactive oxygen species (ROS). A well-studied example is Cu(Phen)_2_ (where Phen = 1,10-Phenanthroline), which cleaves DNA oxidatively from the minor groove (Chen and Greenberg 1998; Bales et al. 2002; Slator et al. 2018). Despite its promise, limitations such as moderate DNA binding affinity, ligand dissociation and dependence on exogenous reductants have prompted the design of improved analogues. To address these challenges ternary Cu(II) complexes incorporating DNA intercalators, such as Cu-DPQ-Phen (Molphy et al. 2014), were developed to enhance DNA binding and cleavage efficiency. Additionally, Cu-Clip-Phen derivatives have been reported, which covalently link the phenanthroline ligands via a serinol linker to maintain the 2:1 ligand-to-metal ratio and reduce ligand dissociation (Pitié et al. 1998). Polynuclear copper complexes, including di-nuclear Cu-Oda (Slator et al. 2018) and Cu-BPL-C6 (Poole et al. 2025), and the tri-nuclear ‘Tri-Click’ series (Kellett et al. 2025; Gibney et al. 2023; McStay et al. 2021) (Fig. 3), were designed to facilitate cooperative DNA interactions and ‘self-activated’ (i.e*.* without co-activating reductants) oxidative cleavage. Two recent examples of these systems include Cu_2_-BPL-C6 and Cu_3_-TC-Thio, which are polynuclear copper complexes synthesised by CuAAC chemistry (Fig. 3a) (Poole et al*. *2025) (Gibney et al. 2023). They exhibit high DNA affinity (~ 10^7^ M^−1^), and NCI-60 cytotoxicity against multiple cancers, with efficient self-activated damage (Melvin et al. 2000). In parallel, there have been several notable reports of ruthenium(II) metal complexes interacting with dsDNA. For example, the ruthenium polypyridyl complex Λ-[Ru(Phen)_2_(11-CN-DPPZ)]^2+^ (Fig. 3a) (Cardin et al. 2017), which binds enantiospecifically to DNA through intercalation, and the site-specific Λ-[Ru(Phen)_2_Phi]^2+^ featuring the Phi ligand (9,10-phenanthrenediimine) demonstrate a remarkable ability to target specific DNA sequences, as evidenced by its preferential intercalation into the DNA decamer duplex d(CCGGTACCGG)_2_ (Prieto Otoya et al. 2024a).

The interaction of copper and ruthenium complexes with dsDNA, their ability to induce conformational changes, and any base-pair specificity has been investigated using numerous biophysical and molecular techniques. Here, we will focus on X-ray crystallography, which provides primary data for the PDB structural database—together with Nuclear Magnetic Resonance (NMR), which has been extensively reviewed elsewhere (Searle 1993; Bhaduri et al. 2018; Kumar et al. 2012)—and hence structure prediction algorithms, combinatorial studies employing circular dichroism (CD), microscale thermophoresis (MST) and FRET (Fluorescence Resonance Energy Transfer) thermal melting analysis, along with recently developed techniques such as in-liquid atomic force microscopy (AFM) and single molecule imaging.

### X-ray crystallography

X-ray crystallography gives precise experimental coordinates for compounds bound to DNA at atomic resolution. The ruthenium polypyridyl complex [Ru(Phen)_2_(11-CN-DPPZ)]^2+^ was studied bound to both duplex and G-quadruplex DNA, revealing its intercalative binding mode to duplex DNA. In this process, the planar dipyridophenazine (DPPZ) ligand inserts between DNA base pairs from the minor groove. The 11-CN substitution on the DPPZ ligand introduces asymmetry, enhancing selectivity for specific DNA sequences, particularly those rich in guanine-cytosine (GC) pairs. Structural data showed that the complex preferentially intercalates at DNA steps such as TC/GA, with the cyano (CN) group projecting into the major groove to optimise stacking interactions with guanine bases. This sequence specificity improves upon non-CN derivatives like [Ru(Phen)_2_(DPPZ)]^2+^, which lack such binding preference. The polar CN group strengthens stacking interactions, contributing to increased binding affinity and enhanced luminescence upon DNA binding, making it valuable for DNA imaging applications. In contrast, [Ru(Phen)_2_Phi]^2+^ exhibits a distinct binding mode with a preference for the major groove of DNA. Crystallographic studies have stabilised that the Λ-enantiomer of [Ru(Phen)_2_Phi]^2+^ intercalates symmetrically into the major groove at the central TA/TA step of the DNA decamer duplex d(CCGGTACCGG)_2_. This binding mode is complemented by angled (canted) intercalation with H-bond stabilisation in the minor groove at adjacent GG/CC steps (Prieto Otoya et al. 2024a).

### Combinatorial studies

To gain a detailed understanding of how metallodrugs interact with DNA, it is important to employ a range of biophysical techniques that provide complementary insights for the complex drug-DNA interactions occurring. A series of DNA hairpins, based on the Dickerson-Drew dodecamer (Dickerson et al. 1982; Wing et al. 1980), where a non-labelled, 5′-fluorophore-labelled and a 5′-fluorophore-3′-quencher derivative were synthesised (Fig. 3b), was to be used in CD, MST and FRET-melting, respectively. Variations of these sequences were also employed, where central AT-rich, GC-rich, AT vs TA steps and TATA binding profiles were investigated (Fig. 3c).

### Circular dichroism spectroscopy 

Circular dichroism (CD) spectroscopy measures the difference in absorption of left and right circularly polarised light by chiral molecules, which provides insights into their structure and conformation. UV–vis CD has been extensively employed to study the secondary structure of biomolecules including A-, B- and Z-DNA. CD can thereby provide insights into drug-DNA interactions, by observing changes in the UV spectrum at specific wavelengths (200–320 nm) (Kellett et al. 2019b). For many years, CD spectroscopy has been performed with long DNA sequences of varying A/T-G/C content, such as calf thymus DNA (ctDNA), poly[d(C-G)_2_] DNA and poly[d(A-T)_2_], but more recently short palindromic DNA oligonucleotides of defined sequence have become increasingly employed (Kellett et al. 2019b). Using these short sequences is advantageous as the interaction of a drug molecule with specific base-pairs or tracts can be investigated, as highlighted for di-nuclear Cu-Oda, where its ability to induce the ‘Z-like’ DNA was elucidated (Slator et al. 2018). To further increase the utility of CD spectroscopy to study drug DNA interactions, recent adaptations in the experimental design of short DNA hairpins were used to probe the interactions of metallodrug candidates (Fig. 3b). For example, Cu_3_-TC-Thio—a ‘Tri-click’ complex synthesised using CuAAC chemistry—was investigated using a Dickerson-Drew hairpin (DDH) sequence (Gibney et al. 2023). It was noted that the CD spectra displayed a decrease in intensity at 250 and 280 nm upon binding. These wavelengths are related to electronic transitions of helicity (245 – 250 nm) and base stacking interactions (275 – 280 nm) (Kypr et al. 2009), indicating that Cu_3_-TC-Thio disrupts the native B-DNA structure, in a dose-dependent manner. Less chiral ordering and less efficient exciton coupling between bases lowers CD intensity, a response that signifies a loss of DNA helicity and base pair stacking, indicating a non-intercalative binding mode consistent with minor groove recognition, and potential condensation effects (Gibney et al. 2023).

### Microscale thermophoresis

MST measures binding affinity and conformational changes by tracking the movement of fluorescently labelled molecules in a temperature gradient, offering quantitative insights into drug-DNA interactions (Seidel et al. 2013). Employing the fluorophore-labelled DDH (F-DDH), the interaction of a binding agent can be probed (Fig. 3b). MST studies with Cu_3_-TC-Thio and F-DDH yielded an EC_50_ value of approximately 70 μM, indicating strong DNA binding and potential condensation effects, which confirmed the condensation profile observed during CD analysis (Gibney et al. 2023). This MST assay design was then further expanded for the analysis of the enantiomers of the ruthenium complex, [Ru(Phen)_2_Phi]^2+^. The sequence-specific binding affinity of the lambda- and delta-enantiomers of [Ru(Phen)_2_Phi]^2+^ were investigated by MST using fluorophore-labelled hairpins with varied sequence compositions (Fig. 3c). The Λ-enantiomer exhibited stronger binding across all sequences, with dissociation constant (*K*_d_) 580 nM with F-DDH and 610 nM with the GC-rich F-D7H, compared with *K*_d_ = 1 μM for Δ-[Ru(Phen)_2_Phi]^2+^, while both enantiomers displayed strong binding interactions with the TATA hairpin (F-D6AH) (*K*_d_ = 520 nM and 620 nM, for Λ- and Δ-[Ru(Phen)_2_Phi]^2+^, respectively). Interestingly, in the presence of a hairpin with GGCC repeats (F-TP), Λ-[Ru(Phen)_2_Phi]^2+^ (*K*_d_ = 430 nm) was a significantly better binder than Δ-[Ru(Phen)_2_Phi]^2+^ (*K*_d_ = 960 nM) underscoring the Λ-enantiomer preference for GGCC repeats, and highlighting its precision as a site-specific metallodrug (Prieto Otoya et al. 2024a). These modified DNA hairpins were also utilised for the analysis of the di-nuclear Cu_2_-BPL-C6—a dinuclear copper complex that was synthesised using copper(I)-catalysed azide-alkyne cycloaddition (CuAAC) click chemistry—which revealed a similar DNA binding affinity to the sequences examined—in the order of 10^6^ M^−1^—together with significant DNA aggregation (Poole et al. 2025).

### FRET thermal melting

The melting temperature (*T*_m_) of a DNA sample corresponds to the equilibrium temperature at which 50% of the DNA is in helical form and 50% is single-stranded (Guedin et al. 2010; Kellett et al. 2019a). The *T*_m_ value can be determined through absorbance or fluorescence spectroscopy, depending on the experimental setup. Nucleic acids display maximal absorbance at 260 nm, which undergoes a hyperchromic shift (i.e*.* an increase in optical density) as DNA is denatured to its single-stranded form (Guedin et al. 2010). Fluorescence melting is an alternative method that typically relies on the bound state of an exogenous fluorophore for detection. Commonly employed fluorophores used to probe DNA include SYBR green and FRET probes. We employed DNA hairpins—identical to those used for CD and MST analysis—labelled with a FRET pair (5′-Alexa Fluor 647 (fluorophore) and 3′-Iowa Black (quencher)) to evaluate how new compounds interact with specific sequences by altering the stability of underlying DNA structure (Fig. 3b). This method was used to characterise the interaction between Cu_3_-TC-Thio and FRET-DDH containing a 5′-Alexa Fluor 647 (F) and 3′-Iowa Black (Q) pair. The native *T*_m_ of the hairpin was 79 °C, and the addition of the copper complex resulted in a significant dose-dependent *T*_m_ increase, demonstrating DNA stabilisation. Analysis of the melting curves provided a binding constant (*K*_b_) of 5.8 × 10^7^ M^−1^ indicating high-affinity binding, and an occupancy of approximately 3.5 molecules per hairpin, revealing the stoichiometry of the interaction (Gibney et al. 2023). Later studies using hairpins of different sequence context revealed that the Λ-[Ru(Phen)_2_Phi]^2+^ enantiomer exhibited greater thermal stabilisation than Δ-[Ru(Phen)_2_Phi]^2+^, with the highest stabilisation (Δ*T*_m_ = + 22.4 °C) found for the TATA hairpin (FRET-D6AH), which demonstrates selective targeting that reinforces the X-ray crystallographic studies (Prieto Otoya et al. 2024a). A final example of this method can be seen during the DNA binding mode characterisation of Cu_2_-BPL-C6. Here, a range of FRET-labelled hairpins (Fig. 3c) were co-incubated with the complex with data showing a stabilising effect with AT-rich hairpins FRET-1 (*r* = 7, where *r* = [drug]/[DNA]; + 4.5 °C) and FRET-2 (*r* = 10, where *r* = [drug]/[DNA]; + 3.6 °C) and an overall destabilising trend with GC-rich hairpins. Further analysis using the Bard equation facilitated the calculation of direct binding (*K*_b_) which was evaluated as 1.06 × 10^7^ M^−1^ and an occupancy of two molecules per hairpin while a *K*_b_ of 4.38 × 10^8^ M^−1^ with an occupancy of three molecules per hairpin was obtained for FRET-2 (Poole et al. 2025).

### In-liquid atomic force microscopy

Atomic force microscopy (AFM) offers high-resolution imaging of DNA structural changes, enabling direct visualisation of a compound’s effects at the molecular level (Engel et al. 1999; Binnig et al. 1986). Traditional AFM is typically performed in air or within a vacuum and requires samples to be dried, which can distort DNA’s natural conformation due to dehydration, leading to artefacts such as strand collapse or aggregation (Holmes et al. 2025; Lyubchenko 2011). In contrast, in-liquid AFM (Tamayo et al. 2001) allows imaging of DNA in its native, hydrated state within aqueous environments like water or buffer solutions, preserving its biological structure and function (Holmes et al. 2025; Main et al. 2021). This technique not only eliminates drying artefacts but also reduces mechanical damage from the AFM probe, as the liquid medium cushions the interaction between the probe and the sample. Furthermore, in-liquid AFM enables real-time observation of dynamic processes, such as DNA conformational changes or drug-induced damage, providing insights into the kinetics of molecular interactions. These advantages make in-liquid AFM particularly valuable for studying DNA interactions with metallodrugs. For example, in-liquid AFM revealed that Cu_2_-BPL-C6 exhibited single-molecule DNA cleavage and pronounced condensation effects (Poole et al. 2025). In-liquid AFM has also been combined with a Topoisomerase I inhibition assay (Kellett et al. 2019b) to evaluate the inhibition of DNA unwinding and DNA condensation when treated with poly-nuclear platinum complexes (triplatinNC and N_3_-triplatinNC) (O'Carroll et al. 2025) This technique has also been used to characterise DNA triplex formation, paving the way for a more detailed assessment of higher-order DNA structures (Pyne et al. 2021). This ability to capture such detailed, physiologically relevant data highlights the superiority of in-liquid AFM over traditional methods for investigating DNA-drug interactions.

Table 2 summarises these methods.

**Table 2:** Techniques used to identify drug-DNA binding with site-specific interactions

Technique	Example application
Circular dichroism (CD) spectroscopy	Monitors conformational changes in DNA structure upon drug binding, such as detection of B → Z DNA transitions by Cu-Oda and identification of groove binding or intercalation (Slator et al. 2018)
Microscale thermophoresis (MST)	Measures direct binding affinity (*K*_d_) of a drug molecule to DNA, such as quantification of binding affinities to reveal sequence preference like preferential binding of metal complexes for GC-rich vs AT-rich sequences (Prieto Otoya et al. 2024a; Gibney et al. 2023)
Thermal melting (*T*_m_) experiments	Detects changes in DNA duplex stability in the presence of a drug, such as evaluation of sequence-specific thermal stabilisation (e.g. enhanced stability of TATA box-containing sequences by metal complexes relative to other motifs) (Poole et al. 2025; Prieto Otoya et al. 2024a)
X-ray crystallography	Enables atomic-level resolution of drug-DNA interactions, such as structural elucidation of binding modes like intercalation with sequence-specific preferences by ruthenium complexes relative to groove binding (Prieto Otoya et al. 2024a; Cardin et al. 2017)
In-liquid AFM	Enables visualisation of DNA topologies in hydrated form, such as detection of DNA triplex formation at base-pair resolution (Holmes et al. 2025; Pyne et al. 2021)

### Targeting duplex DNA with triplex-forming oligonucleotides

**Fig. 4:**
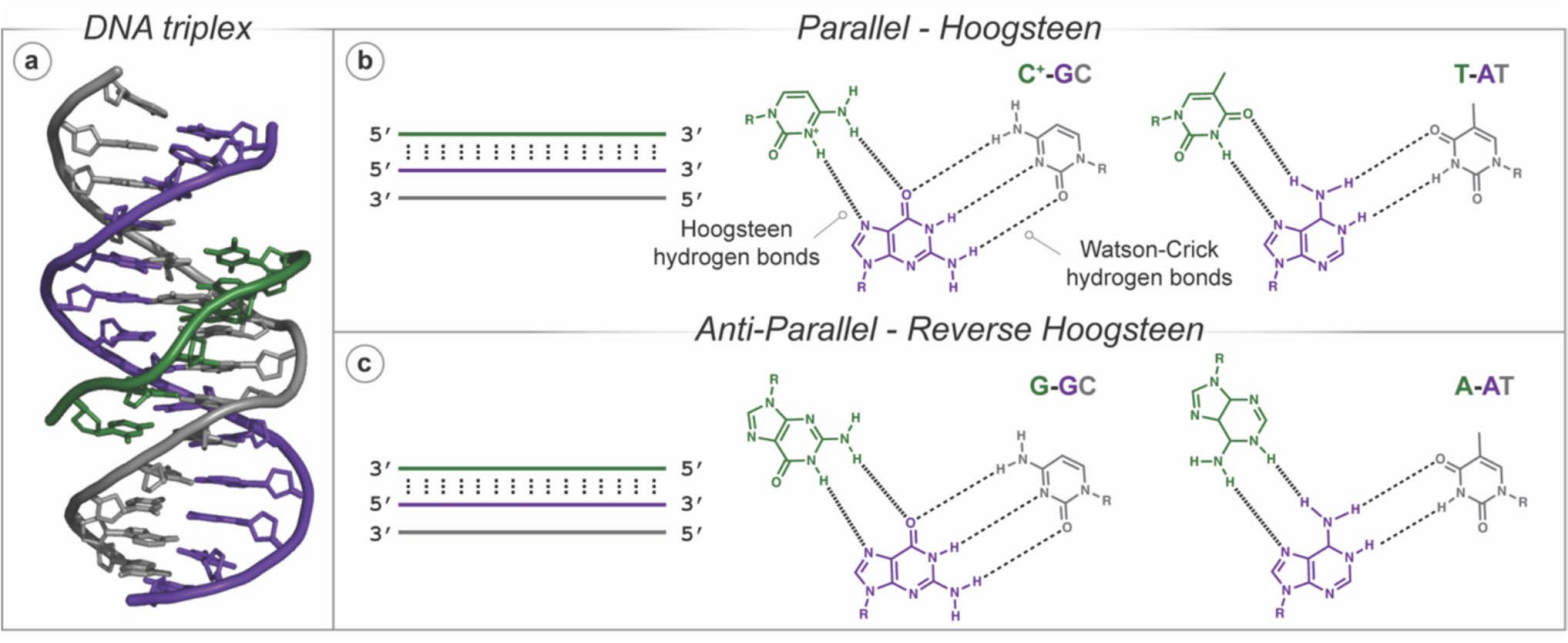
**a** Solution NMR of a parallel DNA triplex (TFO–green) showing Hoogsteen base pairing (PDB: 1BWG); **b** parallel triplex formation with Hoogsteen (T-AT and C^+^-GC) base pairing; **c** anti-parallel triplex with reverse-Hoogsteen (G-GC and A-AT) base pairs. Note: Watson-Crick and Hoogsteen hydrogen bonds are artificially elongated for clarity

DNA triple helices were first reported by Felsenfeld, Davies and Rich in 1957, when it was demonstrated that polyuridylic and polyadenylic acid ribonucleotide strands in a 2:1 ratio could form stable triple helical structures in the presence of divalent cations (Felsenfeld et al. 1957). Next, it was reported that short pyrimidine-rich RNA oligonucleotides could sequence-selectively bind within the major groove of poly-purine dsDNA. In 1979, Lee et al*.* demonstrated that short DNA oligonucleotides could also form triplexes within the major groove of poly-purine/pyrimidine tracts (Lee et al. 1979). Triplex-forming oligonucleotides (TFOs) can be poly-pyrimidine—and bind parallel to the DNA duplex (Fig. 4)—or poly-purine—and bind either parallel or antiparallel (Fig. 4)—but they exclusively bind with the purine-rich strand within dsDNA, as adenine and guanine can form two Hoogsteen hydrogen bonds with the TFO (Knauert and Glazer 2001; Fox et al. 2018). In purine-rich TFOs, these bonds form between A and the AT Watson–Crick pair, while G binds to the GC pair, forming A-AT and G-GC triplets (Beal and Dervan 1991; Durland et al. 1991). Similarly, in pyrimidine TFOs, the T binds to AT, but the C bases must be protonated at N3 to form two Hoogsteen bonds with the GC base pair; thereby the binding motif for pyrimidine TFOs is T-AT and C^+^-GC (Fig. 4) (Moser and Dervan 1987; Le Doan et al. 1987; Lee et al. 1979; Miller and Sobell 1966). Both triplex motifs are capable of recognising and binding to their corresponding duplex sequence, but pyrimidine TFOs—which bind parallel to the target duplex—are isomorphic (i.e*.* if the C1′ atoms of the Watson–Crick base pairs are superimposed, the positions of the C1′ atoms of the TFO are almost identical), which minimises backbone distortion (Thenmalarchelvi and Yathindra 2005; Fox et al. 2018). Additionally, purine TFOs are often capable of self-association into structures such as G-quadruplexes and GA-duplexes; triplex formation competes with this self-association, reducing the TFO concentration available to bind with its target (Fox et al. 2018). Therefore, pyrimidine-rich TFOs are more commonly used but require acidic conditions to protonate the C bases in the TFO strand. This requirement initially limited their functionality as they struggled to form triplexes under physiological conditions. However, many modifications (to the base, sugar and phosphate backbone) that increase the binding stability of CT-rich TFOs have since been reported to enable binding at physiological pH (Fox et al. 2018). TFO modifications have been discussed elsewhere (Fox et al. 2018; Faria and Giovannangeli 2001; Vasquez and Glazer 2002; Fox and Brown 2011; Rusling 2021; Walsh et al. 2018; Dalla Pozza et al. 2022) and it has been noted that using a combination of triplex stabilising agents and/or modified bases enables pyrimidine TFOs to form stable triple helical structures under physiological conditions.

**Fig. 5:**
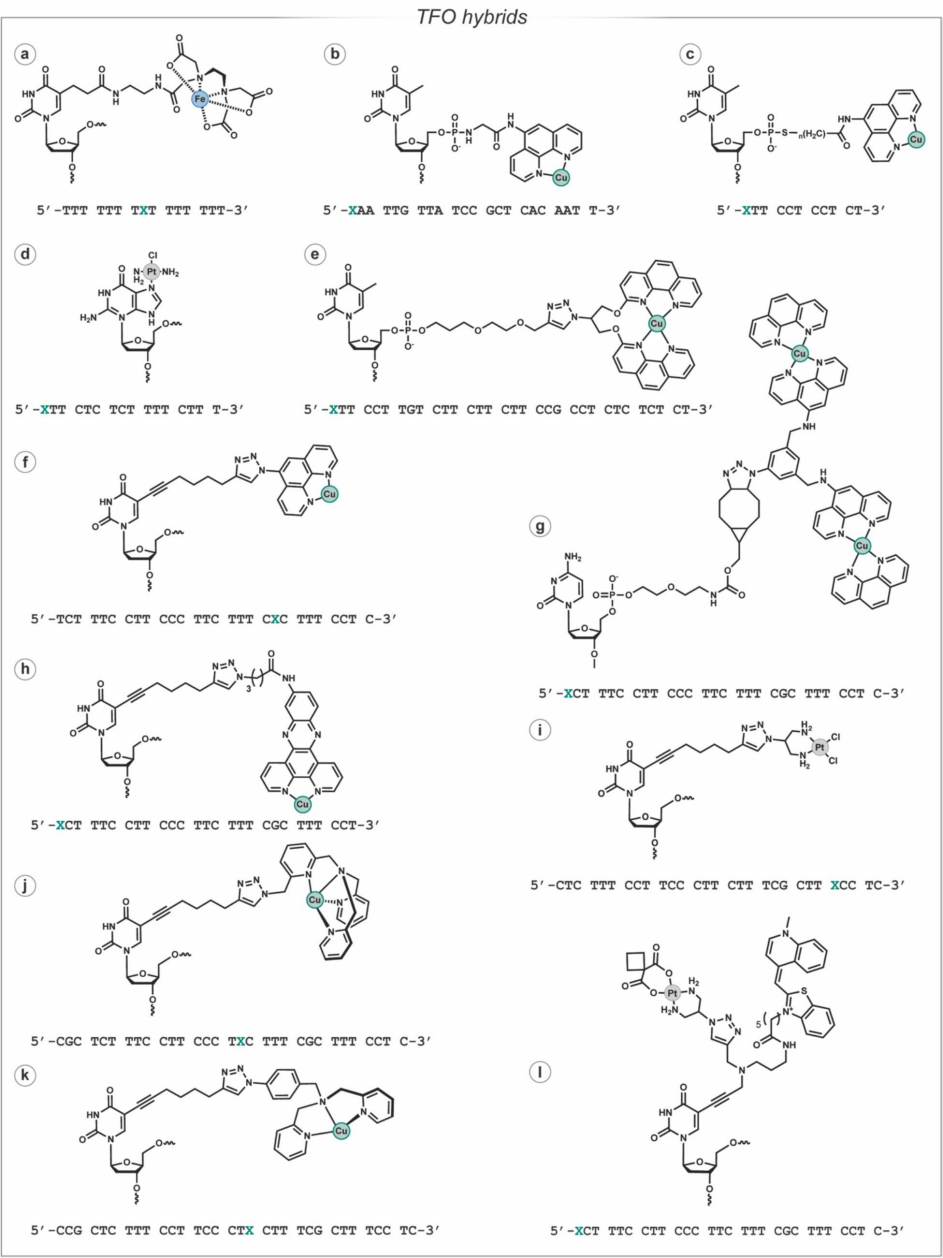
TFO hybrids synthesised through traditional and click chemistry strategies. Position of modification within the TFO sequence is indicated by a green ‘X’ within the sequence shown below the structures of the modified nucleotides

The sequence selectivity of TFOs sparked interest in their utility as anti-gene therapeutics, and TFOs have also been conjugated with various DNA-damaging agents to achieve targeted DNA damage. In 1987, Moser and Dervan described a (Fe-EDTA)-TFO (Fig. 5a) that hybridised with a specific poly-purine sequence and consequently targeted Fe-EDTA cleavage to that sequence (Moser and Dervan 1987). This strategy of attaching DNA-damaging agents to TFOs was also investigated for Cu-Phen (Fig. 5b, c) (Chen and Sigman 1986; François et al. 1989) and platinum derivatives (Fig. 5d) (Graham and Miller 2012; Sharma and McLaughlin 2002), but the complex synthetic protocols required to produce these TFO hybrids limited their applications. Nucleic acid click chemistry (Kolb et al. 2001; Agard et al. 2004; Tornøe et al. 2002; Gierlich et al. 2006) provided an alternative solution for attaching DNA-damaging agents to anti-gene TFOs. In 2020, the first ‘click’-generated artificial metallo nuclease (AMN) TFO hybrid was reported (Panattoni et al. 2020). Click chemistry–synthesised AMN-TFO hybrids have been reported for clip-1,10-phenanthroline (Fig. 5e) (Panattoni et al. 2020), phenanthrene ligands (Phen (Fig. 5f), di-nuclear bis-Phen (Fig. 5g), DPPZ (Fig. 5h) and Phenanthroimidazole) (Lauria et al. 2020), tris-(2-picolyl)amine (TPMA) (Fig. 5j) (Zuin Fantoni et al. 2020) and di-(2-picolyl)amine (DPA) (Fig. 5k) (McGorman et al. 2022). Click-generated *cis*-platinum(II) chemotypes—cisplatin (Fig. 5i), carboplatin (Fig. 5l) and oxaliplatin—have also been reported (Hennessy et al. 2024, 2022), which overcame limitations of the requirement for complexation between a platinum(II) reagent and the TFO.

Analysing the binding affinity and sequence selectivity of TFOs is of great importance, and a wide range of biophysical and molecular biology assays have been reported. This review focuses on thermal melting, gel electrophoresis and NMR as the assays provide important biophysical insights for triplex formation and the stability of triple helical DNA.

### UV and fluorescence thermal melting

Thermal melting has been employed to investigate the stability of triple helical DNA and the impact of modifications included within and attached to the TFO sequence. Absorbance and fluorescence melting are widely used to determine the *T*_m_ of triple helical DNA, where a biphasic—typically TFO followed by dsDNA—melting profile is observed. For TFOs with high *T*_m_, the triplex melt can occur within the same temperature range as the dsDNA, limiting this technique’s utility. However, if an intercalating fluorophore (e.g. thiazole orange) (Walsh et al. 2018) or a FRET pair (Darby et al. 2002) is included in the TFO sequence, the *T*_m_ of the triplex can be monitored—through changes in fluorescence—independent of the dsDNA. The *T*_m_ of the TFO is dependent on the sequence composition, presence and/or number of triplet inversion sites (i.e*.* where a purine interrupts the poly-pyrimidine sequence), ribose and/or base modifications and groups attached to the TFO. For example, UV thermal melting has been employed to evaluate how intercalating (e.g. thiazole orange, phenanthrene ligands), non-covalent (e.g*. *copper caging systems—TPMA and DPA) and covalent (e.g*.* platinum complexes) moieties impact triplex stability. In general, intercalators increase the *T*_m_ of TFOs, while the attachment of other groups (e.g*.* platinum complexes) destabilises triplex formation (McGorman et al. 2022; Hennessy et al. 2022; Zuin Fantoni et al. 2020). Thiazole orange is a particularly efficient stabilising group as the incorporation of a single thiazole orange derivative at the 5-position of thymine can increase triplex stability by ~ 23 °C (pH 5.8) with the addition of a second and third thiazole orange modification increasing triplex melting temperatures above 78 °C (Walsh et al. 2018). It was also reported under physiological conditions (pH 7) that when three bases were modified with thiazole orange, the triplex was stabilised by > 26 °C (Walsh et al. 2018). Thiazole orange has also been utilised within carboplatin-TFOs, where thiazole orange was attached either to an internal nucleobase—independent of carboplatin—or to a specifically designed bifunctional nucleobase (Fig. 5l) that enables attachment of carboplatin and thiazole orange to the same base (Hennessy et al. 2024). Fluorescent thermal melting, with thiazole orange as the fluorophore, enabled the accurate analysis of the stability of these TFO hybrids across a pH range, highlighting the utility of this fluorescent melting technique.

### Gel electrophoresis

Gel electrophoresis is widely applied for the visualisation of triplex DNA and to evaluate the impact of TFO hybridisation with its dsDNA target. This electrophoretic mobility shift assay (EMSA) (Fried and Crothers 1981; Garner and Revzin 1981) enables the binding of a compound to a DNA sequence of interest to be evaluated. Therefore, EMSA can be adapted to visualise triple helical DNA as a TFO binding to dsDNA increases the molecular mass, and thus the triplex will migrate slower through the gel. However, as non-modified pyrimidine TFOs require a slightly acidic pH to form triplexes at physiologically relevant temperatures, the gel conditions for this EMSA-type assay deviate from standard PAGE conditions. Standard electrophoresis buffers, TBE (Tris–Borate-EDTA) and TAE (Tris–Acetate-EDTA) at pH 8.3, are not compatible with triplex visualisation at room temperature, but tris acetate (TA) buffer at pH 6.1 enables the TFO, dsDNA and triplex DNA to be visualised and their migration compared (Lauria et al. 2020; Zuin Fantoni et al. 2020). However, the usefulness of these conditions was limited due to the ‘frowning’ profile within the gel, which was alleviated through further optimisation and the inclusion of 75 mM NaCl and 5 mM MgCl_2_ (McGorman et al. 2022; Hennessy et al. 2022). Sequence selective cleavage was evaluated using these conditions, where the damage inflicted on target dsDNA was visualised and compared to an *off*-target control (McGorman et al. 2022). Multiplex gel analysis of targeted platinum(II) adducts and their subsequent removal with sodium cyanide was also compatible with these EMSA conditions, while denaturing 1X TBE PAGE enabled the strand on which the platinum adducts formed to be determined (Hennessy et al. 2022).

Cleavage products resulting from incubating AMN-TFOs with their dsDNA target have also been evaluated through footprinting assays, where sequence selective cleavage products are compared to DNA fragments of known size. This has been achieved using a-^32^P radiolabelled (Panattoni et al. 2020) and fluorophore-labelled (Lauria et al. 2020) DNA, where intact dsDNA controls and the cleavage fragments were visualised. Fluorophore labelling is advantageous, as each strand of dsDNA can be independently visualised, which enables a more precise analysis of the damage inflicted (Lauria et al. 2020). DNase-1 footprinting is an alternative method, where the DNase-1 enzyme digests dsDNA, but the presence of a DNA binding ligand protects the bound region of DNA from digestion (Hampshire et al. 2007). This technique has been employed to evaluate the triplex stability of native and modified sequences (Rusling 2021). The hybridisation of a TFO prevents digestion within its binding site, and by visualising DNase-1 footprinting at varied pH, the improved triplex stability afforded by the Z base was determined (Rusling 2021).

### Nuclear magnetic resonance

NMR enables evaluation of the structure and dynamics of DNA triplexes in a solution-state environment. Triplex structures can be characterised by NMR through the analysis of chemical shifts, Nuclear Overhauser Effect Spectroscopy (NOESY) correlations, and scalar couplings (Kaushik et al. 2019; Feigon et al. 1995). The presence of Hoogsteen and reverse Hoogsteen hydrogen bonds, which are characteristic of DNA triplexes, leads to unique imino proton chemical shifts that are distinct from those of standard Watson-Crick base pairs (Kan et al. 1991). NOESY experiments are crucial as they reveal through-space correlations between nuclei that are close to each other, allowing researchers to determine the three-dimensional fold of the triplex and confirm the non-canonical base pairing. While NMR has been widely used to study DNA triplexes in vitro, it has also been used for in-cell NMR studies, providing direct evidence that these structures can form inside living cells (Sakamoto et al. 2021). More recently, NMR has been employed to investigate triplex formation with dsDNA and an LNA TFO, where it was found that the structure of the dsDNA duplex changes to accommodate the LNA strand where the dsDNA adopts a geometry intermediate between A- and B-type DNA (Sørensen et al. 2004). Table 3 summarises these methods.

**Table 3:** Techniques employed to analyse DNA triplexes

*Technique*	*Example application*
UV thermal melting	Investigates the stability of triple helical DNA and evaluates factors impacting stability such as sequence composition, triplet inversion sites and stabilising agents (Hennessy et al. 2022; Zuin Fantoni et al. 2020; Lauria et al. 2020)
Fluorescence thermal melting	Monitors the *T*_m_ of a DNA triplex, using a fluorescent dye (e.g. SYBR green) (McGorman et al. 2022) or can investigate the stability of the TFO independent of dsDNA using an intercalating fluorophore (e.g. thiazole orange) or FRET pair incorporated into the TFO sequence (Walsh et al. 2018; Hennessy et al. 2024)
Gel electrophoresis	Provides biophysical insights into triplex formation and the stability of triple helical DNA, assessing sequence selectivity and binding affinity of TFOs (McGorman et al. 2022; Hennessy et al. 2022; Rusling 2021)
NMR	Evaluates the structure and dynamics of triplexes in a solution-based environment (Kaushik et al. 2019; Sørensen et al. 2004; Feigon et al. 1995; Kan et al. 1991)

### Three-way junctions

**Fig. 6:**
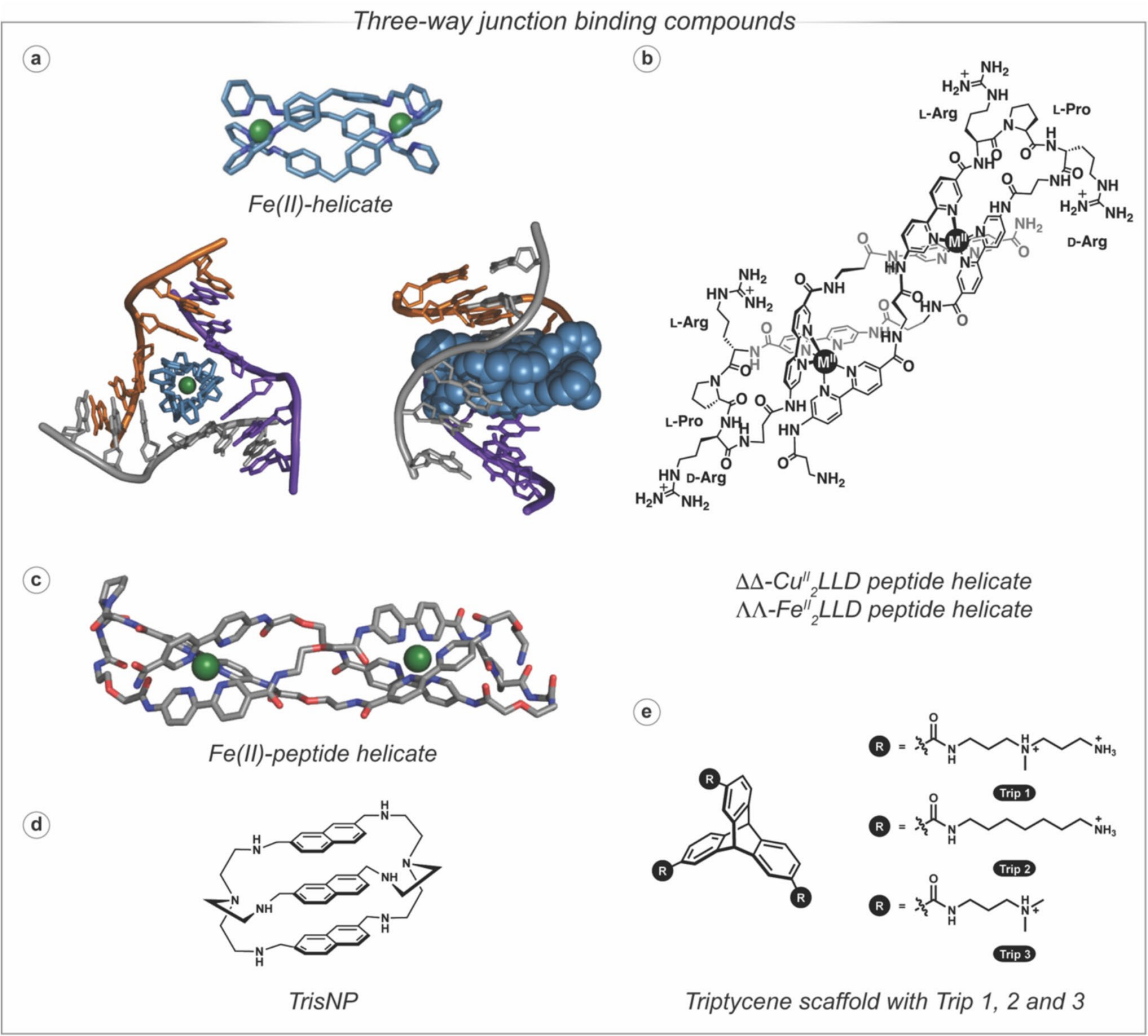
**a** Structure of the Fe(II) metallosupramolecular helicate assembly, with top-down and side-on binding with 3WJ DNA (PDB: 2ET0); **b** the molecular structure peptide helicate that can bind Cu(II) or Fe(II) (ΔΔ-Cu^II^_2_LLD and ΛΛ-Fe.^II^_2_LLD, respectively), where the Cu(II) derivative can specifically cleave 3WJ DNA; **c** structure of the 3WJ-stabilising iron(II) helicate; **d** structure of TrisNP, an azacryptand with 3WJ-binding properties; and** e** triptycene derivatives Trip 1, Trip 2 and Trip 3

DNA three-way junctions (3WJs) are the simplest non-canonical DNA structure, and they arise as transient intermediates during DNA replication. They also play a role in DNA repair processes triggered by base lesions or single-stranded nicks, as elucidated by structural studies of replication fork reversal mechanisms involving proteins like RecG (Singleton et al*. *2001). Their biological significance is highlighted by their involvement in DNA replication and their presence in triplet repeat expansions linked to genetic disorders, making them promising targets for drug development (Pearson 2002). Consequently, a number of novel compounds have been designed to selectively bind 3WJs (McQuaid et al. 2022), including ligand- and peptide-based iron(II) supramolecular helicates (Gamba et al*. *2014; Gómez‐González et al. 2018), Fe(II) helicates (Fig. 6) (Oleksi et al*. *2006), copper-based helicates (Alcalde-Ordonez et al*. *2023), azacryptands (Novotna et al. 2015; Pipier et al. 2024) and three-fold symmetric triptycene derivatives (Barros and Chenoweth 2014). This section explores several 3WJ-targeting compounds and discusses some diverse biophysical and biochemical methods such as X-ray crystallography and thermal melting, employed to identify and characterise interactions with 3WJs.

### Thermal melting

Thermal melting was employed to evaluate the ability of triptycene-based ligands, developed by Barros and Chenoweth to stabilise 3WJs (Barros and Chenoweth 2014). These triptycene-based ligands—specifically Trip 1, Trip 2 and Trip 3—are built on a threefold-symmetric core and use non-planar π surfaces for shape-selective 3WJ binding, avoiding intercalation with other DNA structures. Significant Δ*T*_m_ stabilisation values of 28.5 °C, 26.3 °C and 18.5 °C, for Trip 1, Trip 2 and Trip 3, respectively, were reported, where Trip 1, with its extended linker and additional amine groups, provided the most substantial effect (Barros and Chenoweth 2014).

Recent research has also introduced metal-induced 3WJ formation and stablisation, with Takezawa et al. demonstrating Ni(II)-stabilised 3WJs using Phen-modified oligonucleotides (Takezawa et al. 2023). Upon addition of Ni(II), which coordinates within the Phen ligands and forms an interstrand [Ni(Phen)_3_]^2+^, the 3WJ is stabilised and this was evaluated through thermal melting experiments (Takezawa et al. 2023). The Phen-modified 3WJ, comprising strands L1, L2 and L3, reported *T*_m_ of 52.8 °C in the absence of metal ions. However, adding one equivalent of Ni(II) ions raised the *T*_m_ to 69.7 °C, yielding a Δ*T*_m_ of + 16.9 °C. The study also compared this to a bipyridine (bpy)-modified 3WJ, which showed a similar Ni^2+^-dependent stabilisation (+ 17.7 °C). Building on the concept, a novel metallo-supramolecular cylinder was developed that exhibits enzyme-responsive binding to 3WJs (Karmakar et al. 2025). In its initial, capped form, the cylinder [Ni_2_(L2)_3_]^4+^ did not significantly alter the melting temperature of 3WJ DNA (*T*_m_ = 40.2 °C versus 39.7 °C for the native 3WJ). However, upon activation by the human enzyme NQO1, which cleaves the six quinone-containing arms, the resulting uncapped cylinder [Ni_2_(L2)_3_]^4+^ binds strongly to the 3WJ, increasing the *T*_m_ to 58.1 °C, yielding a Δ*T*_m_ of + 18.4 °C. Overall, the cylinder’s ability to stabilise the DNA junction upon enzymatic activation offers a unique, switchable approach to modulating DNA structures. While the overall stabilisation effect was less than that of Trip 1 (Δ*T*_m_ = 28.5 °C), the enzyme-responsive nature of the metallo-cylinder provides a level of control, potentially enabling targeted activation in specific cellular environments where NQO1 is overexpressed, such as in specific cancers (Karmakar et al. 2025).

FRET-thermal melting has also been performed to evaluate the 3WJ stabilisation and selectivity properties of azacryptands (Fig. 6d). 3WJ-forming strands were labelled with a 5′-FAM and 3′-TAMRA as the FRET pair, where the quenching of FAM fluorescence and increase in TAMRA fluorescence was indicative of 3WJ formation (Pipier et al. 2024). This study revealed that five of the 15 azacryptands evaluated stabilised 3WJ DNA by > 15 °C, while two of these ligands have limited interactions with a dsDNA control. Competitive FRET studies were also performed to determine the selectivity factor (^FRET^S) for azacryptands (3WJ vs dsDNA and G4s), and it was noted that three of the ligands tested had excellent selectivity (^FRET^S ≥ 90%) for 3WJ, when compared with dsDNA and an additional three had excellent selectivity when co-incubated with 3WJ and G4 DNA (Pipier et al. 2024).

### Crystallography

X-ray crystallography offers detailed structural insights into drug-DNA interactions at the atomic level. The iron(II) metallosupramolecular helicate Fe_2_L_3_Cl_4_ was characterised using this technique to study its binding to a 3WJ formed by the palindromic DNA hexanucleotide 5′-d(CGTACG)−3′ (Oleksi et al*.* 2006). The crystallographic data revealed that Fe_2_L_3_Cl_4_ occupies the hydrophobic cavity of the Y-shaped junction, engaging in electrostatic attractions between the Fe(II) and DNA phosphates, π-π stacking with base pairs, hydrogen bonding and minor groove interactions (Oleksi et al*. *2006). Additionally, enantiomers of supramolecular cylinders were crystallographically examined, demonstrating enhanced 3WJ recognition and potential for constructing extended 3D DNA lattices (Boer et al*.* 2010).

### Fluorescence techniques

Fluorescence-based methods provide quantitative data on binding affinities between compounds and DNA. Fluorescently labelled iron(II) helicates, such as ΛΛ-[Fe_2_(LL-RhH)]^4+^ and ΔΔ-[Fe_2_(DD-RhH)]^4+^, were designed with β-turn-promoting residues like -[(D/L)-Pro]-Gly-, and adopt a di-nuclear hairpin structure. They were examined using fluorescence anisotropy, revealing a 1:1 binding mode to 3WJs with the lambda isomer, ΛΛ-[Fe_2_(LL-RhH)]^4+^, exhibiting a ~ 150-fold higher affinity for 3WJs than its delta counterpart, ΔΔ-[Fe_2_(DD-RhH)]^4+^, underscoring chirality-dependent affinity differences (Gómez‐González et al. 2018; Gamba et al*.* 2014).

Fluorescence quenching has been employed to investigate the binding affinity of peptide helicates. Fluorescein-labelled 3WJ was utilised to investigate the 3WJ binding affinity of a peptide helicate inspired by the viral C_3_-symmetric β-annulus motif (Gómez-González et al. 2021a, b). A 2:3 Fe(II)-to-peptide ligand ratio was found to have 1:1 binding with the 3WJ, and an apparent dissociation constant (*K*_d_) of 308 ± 60 nM was obtained. This assay has also been employed to investigate the binding affinity of peptide ligands that fold into chiral helicates in the presence of labile metal ions, such as Fe(II), Co(II) and Cu(II). Fluorescein-labelled 3WJ was incubated with increasing concentrations of ΛΛ-Fe(II)_2_LLD (LLD = l-Arg-l-Pro-d-Arg) or ΛΛ-Co(III)_2_LLD (Co(II) is oxidised to Co(III) to yield a kinetically inert complex), and quenching of the fluorescein emission was observed at 1:1 for the Fe(II) derivative (*K*_d_ = 0.49 μM and 7.9 μM, for the Fe(II) and Co(III) derivatives, respectively) (Gómez‐González et al*.*, 2021a, 2021b). Later, the copper(II) peptide helicate ΔΔ-Cu(II)_2_LLD was analysed using fluorescence quenching, confirming high-affinity binding to 3WJs (*K*_d_ = 260 nM) (Alcalde-Ordonez et al*.*). Recently, Vázquez López and coworkers have further expanded this assay to evaluate the 3WJ-binding properties of self-assembling chiral Co(II) Peptide Helicates Derived of BTMA-1. They reported that the chiral aggregate (P-agg)—formed by BTM-1 in the absence of metal ions—can act as a reservoir of BTMA-1 ligands and enable the in situ assembly of the ΛΛ-Co(II)_2_BTMA-1 helicate in the presence of Co(II) ions and the recognition of 3WJ—indicated by fluorescence quenching—in one pot (Alcalde-Ordóñez et al. 2025).

### Gel electrophoresis

Gel electrophoresis is an effective technique to visualise the formation and stability of 3WJs in the presence of binding compounds. In this method, DNA strands are labelled with multiple fluorophores—such as 6-fluorescein (FAM), 6-carboxy-X-rhodamine (ROX) and cyanaine-5 (Cy5)—to enable multiplex analysis. This approach allows for simultaneous detection of different DNA components within a single gel, enhancing the clarity of the results (McGorman et al. 2023). For example, when 3WJ DNA was incubated with ΛΛ-Fe(II)_2_LLD peptide helicate and analysed via native PAGE, a distinct pink-yellow band emerged, indicating junction formation due to the overlay of fluorescent signals (Gómez-González et al*.* 2021a, 2021b). At higher concentrations of the helicate, the intensity of this band diminished, suggesting condensation—a phenomenon consistent with compound-induced aggregation. PAGE was also utilised to examine the hybridisation products and explore the Ni(II)-triggered structural shift between DNA duplexes and 3WJs (Takezawa et al. 2023). Without Ni(II) ions, the Phen-modified strands (L1, L2, L3) and their unmodified complementary strands (S4, S5, S6) formed duplexes, evidenced by a single band on the gel. In the presence of Ni(II) ions, two 3WJs (L1-L2-L3:Ni(II) and S4-S5-S6) were anticipated, though the gel showed no notable changes, indicating a need for further sequence refinement to achieve effective structural induction. However, when mismatch containing duplex strands (M4, M5, M6) were incubated with the Phen-modified strands (L1, L2, L3), dsDNA was observed in the absence of Ni(II), but in the presence of Ni(II) an 88% conversion to 3WJ was observed. This indicated that the formation of the 3WJs is due to induced interstrand complexation of the Ni(II)(Phen)_3_ (Takezawa et al. 2023).

### Microscale thermophoresis

MST is employed to quantify the binding affinity between compounds and 3WJs with high precision. This technique measures the thermophoretic movement of fluorescently labelled DNA—here, 3WJ with one strand bearing a 5′ Cy5—in a temperature gradient that changes upon compound binding. To perform MST, a serial dilution of ΛΛ-Fe_2_LLD was prepared in an optimised buffer, and Cy5-3WJ was added. A key challenge encountered was DNA condensation at higher helicate concentrations, which was overcome through the addition of a non-ionic surfactant, and the dissociation constant (*K*_d_) for ΛΛ-Fe_2_LLD with 3WJ DNA was calculated (3.033 × 10^−8^ M) (McGorman et al. 2023). Control experiments further demonstrated helicate selectivity, as binding to Cy5-labelled double-stranded DNA (dsDNA) occurred only in the absence of 3WJ, with a *K*_d_ of 3.743 × 10^−6^ M. In contrast, no dsDNA binding was detected when non-fluorescent 3WJ was present, signalling a preference for 3WJs.

### Quantitative polymerase chain reaction stop assay

The quantitative polymerase chain reaction (qPCR) stop assay was first reported as a method to identify DNA regions in *Schizosaccharomyces pombe* that fold into G4 structures, where the presence of a G4 stabiliser inhibited DNA amplification by PCR (Jamroskovic et al. 2019). Pipier et al. recently adapted this qPCR stop assay to investigate the 3WJ stabilisation properties of a family of azacryptands (Pipier et al. 2024). If amplification of a 3WJ-forming sequence is halted, in the presence of an azacryptand ligand, during the qPCR reaction, it indicates that the ligand present stabilises 3WJs and prevents polymerase read-through. The influence of the azacryptands on the fold change in DNA was calculated for sequences containing a 3WJ, a G4-forming region and a scrambled sequence that does not form any non-canonical nucleic acid structures. This enabled the authors to investigate the specificity and binding stability of the ligands for 3WJ DNA, where the naphthalene-based TrisNP-*ana* azacryptand was found to have the highest stability and selectivity for 3WJ DNA (Pipier et al. 2024). 

### Nuclear magnetic resonance

NMR has been widely employed to elucidate the structural details of DNA-ligand interactions, including the formation andstabilisation of 3WJs. Cerasino et al. used this technique to investigate the binding of a tetracationic dinuclear iron(II)supramolecular helicate to palindromic oligonucleotides, demonstrating that the helicate induces a symmetric 3WJ structure insolution. In their study, 1D and 2D NMR techniques, including NOESY and TOCSY, were applied to monitor changes in protonresonances on DNA bases upon titration of the helicate into solutions of d(TATGGTACCATA)_2_ and d(CGTACG)_2_ (Cerasino et al. 2007). The lifting of the helicate’s 2-fold symmetry while retaining 3-fold symmetry around the helicate axis, combined with a 1:3helicate/DNA stoichiometry inferred from 1D spectra, confirmed the assembly of three identical double-helical arms around thehelicate core. NOE contacts between the helicate’s phenyl protons and DNA bases, signalled by significant downfield shifts (1–2ppm), indicated close stacking interactions within a hydrophobic 3-fold symmetric cavity, validating the 3WJ model derived frommolecular dynamics and corroborated by X-ray crystallography. 

Table 4 summarises these methods.

**Table 4:** Methods utilised to characterise 3WJ interactions

*Technique*	*Example Application*
Thermal melting	Investigate thermal stabilisation of 3WJs in the presence of junction-binding ligands (Pipier et al. 2024; Barros and Chenoweth 2014; Takezawa et al. 2023)
X-ray crystallography	Provides detailed insights into binding via electrostatic interactions, π-π stacking, H-bonding and minor groove contacts in the 3WJ cavity (Oleksi et al. 2006; Boer et al. 2010)
Fluorescence techniques	Evaluate binding affinity of a ligand with 3WJ DNA by quantifying fluorescence quenching, the 3WJ or ligand can be fluorescently labelled (Alcalde-Ordonez et al. 2023, Gómez-González et al. 2021a, b, Gamba et al. 2014)
Gel electrophoresis	Visualise 3WJ formation, stabilisation and degradation in the presence of junction-binding ligands (McGorman et al. 2023; Alcalde-Ordonez et al. 2023; Takezawa et al. 2023)
Microscale thermophoresis	Direct quantification of binding with 3WJ DNA. Fluorophore-labelled DNA or ligand is required (McGorman et al. 2023)
qPCR	Determine if ligand (*e.g.* Azacryptands) can block polymerase progression at 3WJ sites (Pipier et al. 2024)
NMR	Monitor changes in proton shifts of DNA bases in a 3WJ which is indicative of compound association/binding (Slavkovic et al. 2024; Cerasino et al. 2007)

### Holliday junction DNA

**Fig. 7:**
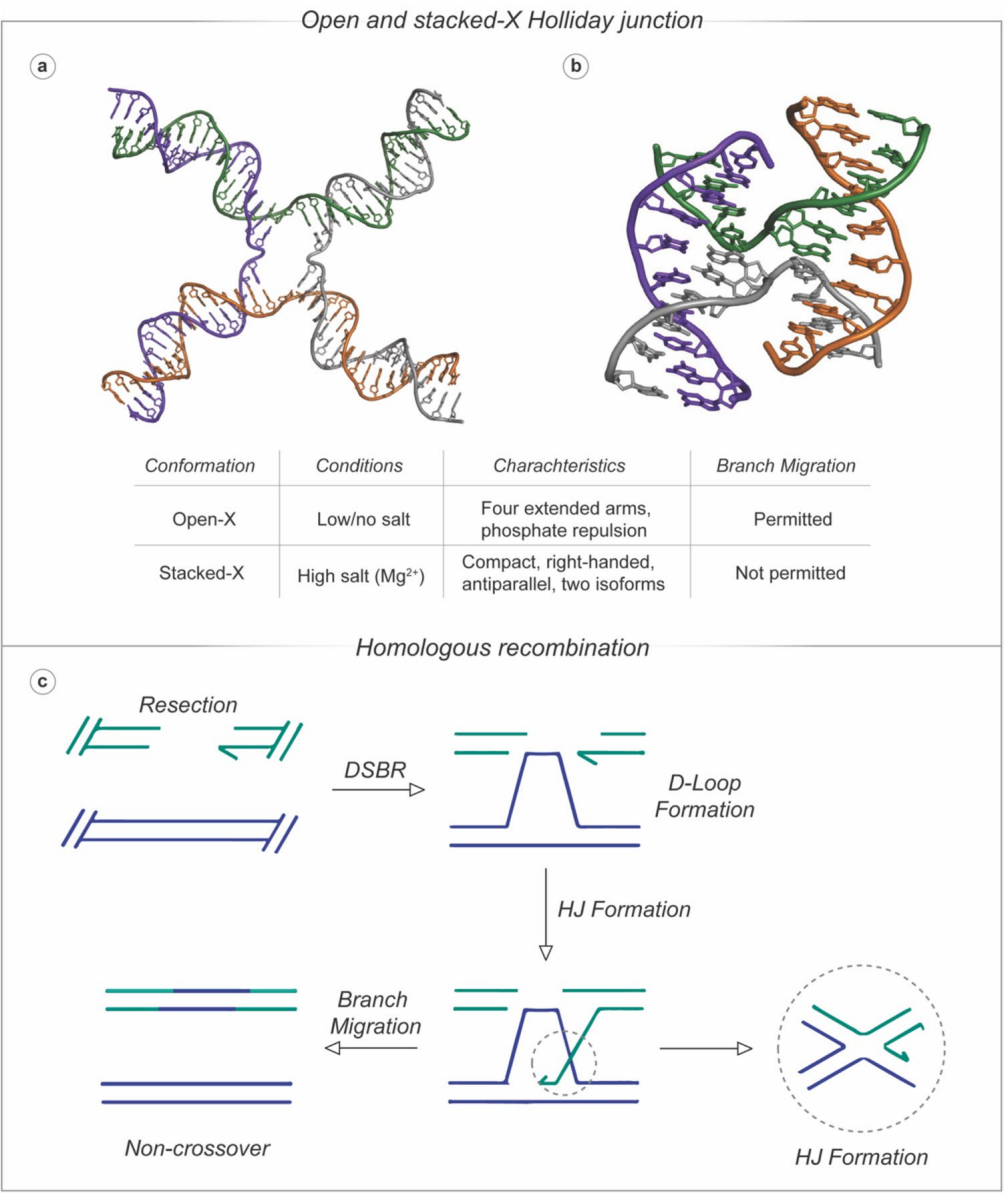
**a** Open-X; and **b** stacked-X conformations of the HJ (PDB: 3CRX, 1DCW); **c** double-strand break repair (DSBR) schematic via homologous recombination

The Holliday junction (HJ) is a non-canonical, sequence-dependent four-way junction that was first described by Robin Holliday in 1964 (Holliday). The structure comprises four nucleic acid strands, and plays essential roles in DNA regulatory functions, such as homologous recombination (HR) and repair. The DNA repair mechanism for DSBs is critical for maintaining genomic stability, a process primarily mediated by HR. Initially, the broken DNA strands undergo resection (Fig. 7b) where nucleases process the DSB to generate single-stranded DNA (ssDNA) overhangs, preparing the DNA for subsequent repair processes (Bizard and Hickson 2014). Following resection, these overhangs facilitate D-loop formation through strand invasion, where one DNA strand invades a homologous template, creating a looped intermediate structure, the synapsis stage of HR (Li and Heyer 2008). The D-loop forms a HJ that enables branch migration and potential genetic exchange (Ranjha et al. 2018). The HJ is then resolved, leading to either crossover or non-crossover products, restoring the integrity of the genome, which is vital for preventing chromosomal aberrations (Segal et al*.* 1997). HJ formation is involved in a number of important biological processes, such as the alternative lengthening of telomeres (ALT) pathway (Haider et al. 2018) and poly(ADP-ribose) (PARP) enzymatic activity (Fábián et al. 2024), which makes it an attractive target in chemotherapeutic development.

HJs exhibit two main conformations: the open-X form, prevalent in low or no salt conditions, with extended arms facilitating branch migration (Lilley 2000; Khuu et al. 2006; Declais 2003) and the stacked-X form, dominant in high salt—particularly Mg^2+^—environments, featuring a compact, antiparallel structure with isoforms (iso I and iso II) that prevent migration (Fig. 7a) (Lilley 2000; Khuu et al. 2006; Declais 2003; Duckett et al. 1988; Murchie et al. 1989; Nowakowski et al. 1999; Ortiz-Lombardía et al. 1999; Eichman et al. 2000; McKinney et al. 2003). HJ formation and stability hinge on specific sequences, such as the ACC core in d(CCGGTACCGG), which enhances stability (~ − 4 kcal/mol), while sequences such as d(CCGCTAGCGG) with an AGC core favour B-DNA (Eichman et al. 2000, 2001; Hays et al. 2003, 2005, 2006; Ho 2017). Sequences with a Pu-Py-C core (e.g*.* d(CCnnnN6N7N8GG)) also promote HJ formation via enhanced hydrogen bonding (Eichman et al*. *2000, 2001*; *Hays et al*. *2003, 2005, 2006; Ho 2017). Environmental factors, including pH, proteins (*e.g.* T7 endonuclease I) and nucleobase modifications (e.g*.* 5-methylcytosine), further modulate HJ dynamics (Declais, Wang et al. 2016).

**Fig. 8:**
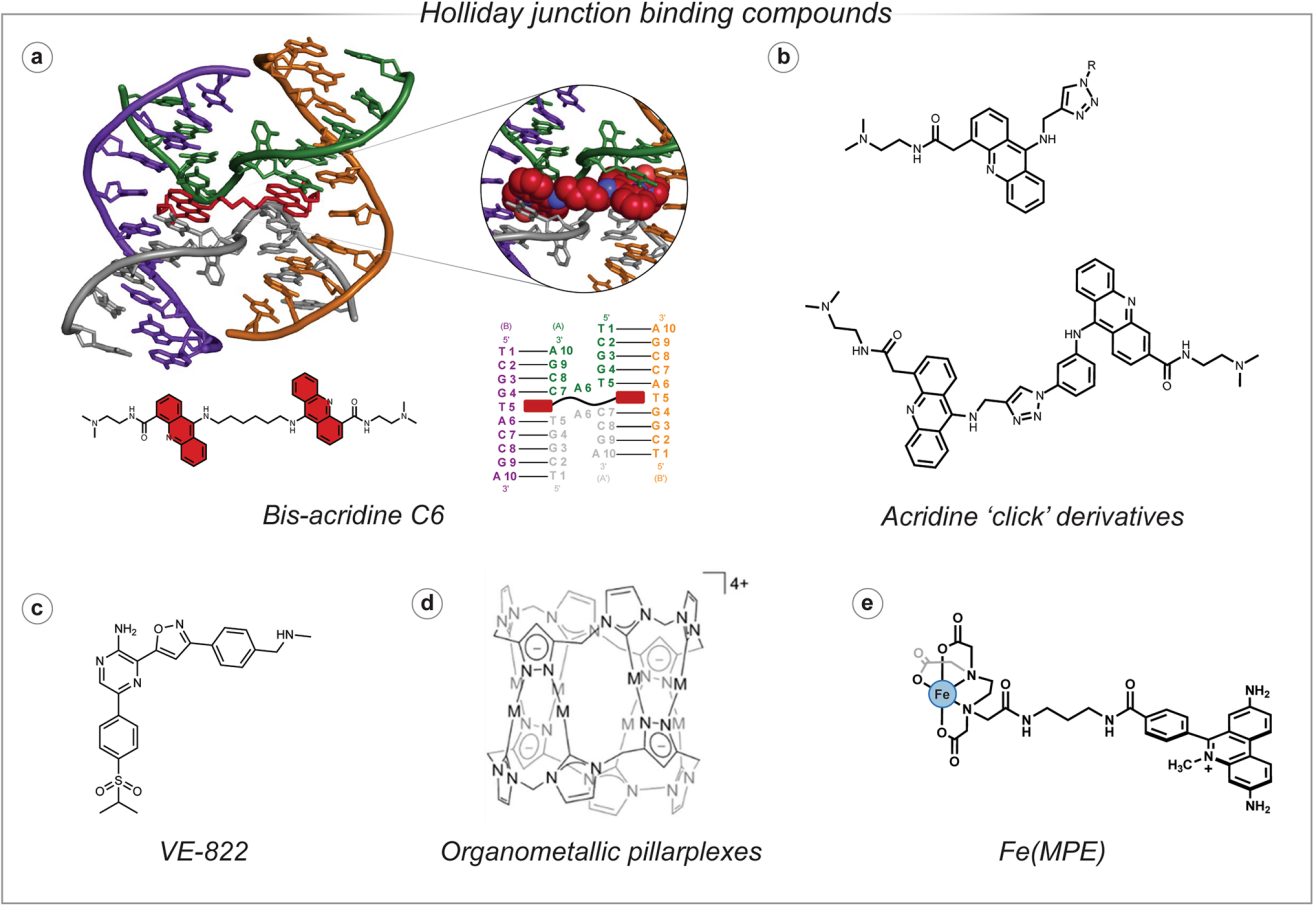
Holliday junction binding compounds; **a** C6 linked bis-acridine ligand (PDB: 2GWA);** b** acridine derivates synthesised using click chemistry; **c** VE-882; **d** organometallic pillarplexes; and **e** Fe(MPE) iron(II) complex

HJ-binding compounds (McQuaid et al. 2022) are essential for probing DNA repair mechanisms and by inhibiting HJ resolution, these agents can induce genomic instability and apoptosis, exemplified by VE-822’s efficacy in enhancing osteosarcoma treatment (Yin et al. 2021). Diverse compounds, including [Fe(MPE)] (Dervan 1986), bis-acridine ligands (Fig. 8) (Brogden et al*. *2007), click chemistry-derived acridine derivatives (Howell et al*.*^2011^, ^2012^), HJ-binding peptides (Cassell et al. 2000 Boldt et al. 2004 Pan et al. 2006), small molecule inhibitors (Ranjit et al. 2010*,* Rideout et al. 2011) and organometallic pillarplexes (Craig et al*. *2023), interact with HJs through mechanisms such as DNA cleavage, structural stabilisation and conformational modulation. These interactions are elucidated using a suite of advanced techniques, including gel electrophoresis, X-ray crystallography, fluorescence assays, molecular dynamics simulations, optical tweezers, atomic force microscopy, bio-layer interferometry, thermal melting, circular dichroism spectroscopy and microscale thermophoresis. These methods collectively provide a detailed understanding of binding affinities, structural dynamics and the functional implications of HJ-compound complexes.

### Gel electrophoresis

Polyacrylamide gel electrophoresis (PAGE) is employed to visualise interactions between HJ DNA and potential binding agents. We recently described a protocol where each strand of HJ DNA was labelled with fluorophores (Alexa Fluor 350 (AF350), FAM, ROX and Cy5) that do not have overlapping fluorescence emission profiles, which enables independent visualisation of each strand of the junction and understanding of the pseudo-duplexes that may form (McGorman et al*.* 2023). This assay was employed to demonstrate that bis-acridine C6 (BA-C6) interacts with the HJ, evidenced by the overlap of fluorescent emission of AF350, FAM, ROX and Cy5, and an upward shift in the gel. This suggests that BA-C6 stabilises the HJ structure through intercalation between the DNA base pairs, which enhances its stability—and gel visibility—and compacts the HJ structure (McGorman et al*. *2023). At higher concentrations, it was noted that the bands disappear entirely, indicating aggregation and the formation of larger DNA-ligand complexes. Howell et al*.* also employed PAGE to evaluate a library of click chemistry–derived acridine compounds; they found that a 9-aminoacridine derivative promoted HJ assembly at a concentration of 50 μM (Howell et al*. *2012). This stabilisation outperformed the effect of 2 mM Mg^2+^ ions, implying that this 9-aminoacridine derivative can maintain HJ integrity under conditions where it would typically dissociate, potentially offering a tool to manipulate DNA recombination processes. PAGE was also utilised to reveal that [Fe(MPE)] (Fig. 8e), a metalloporphyrin, increased cleavage efficiency by 60–100% at residues near the HJ branch point compared to duplex DNA (Guo et al*. *1989). This heightened susceptibility to reactive oxygen species (ROS)–mediated damage and highlights the HJ’s unique geometry as a target for complex-induced disruption. Additionally, Craig et al. observed that gold-based pillarplex compounds (Fig. 8d) induced band shifts, suggesting binding to the open-X conformation of HJ DNA (Craig et al*. *2023). At higher concentrations, these compounds rearranged the HJ into Y-shaped forks, indicating a profound structural transition that could influence DNA repair or recombination pathways. Kepple et al. used gel mobility shift assays to demonstrate that peptides WRWYCR and KWWCRW bind to HJ DNA and prevent the binding of the RecG helicase, indicating that these peptides act as competitive inhibitors of RecG by directly interacting with the HJ substrate (Kepple et al. 2008). It was demonstrated that at low peptide concentrations (0.025 μM), RecG binding to HJ DNA was strongly inhibited, with near-complete inhibition at 0.1 μM peptide, while higher RecG concentrations required proportionally more peptide for inhibition, confirming a competitive binding mechanism. Whilst gel electrophoresis is cost-effective and ideal for detecting major structural alterations, its qualitative nature limits its ability to provide detailed insights into subtle binding affinities or kinetic parameters, necessitating complementary techniques for comprehensive analysis.

### X-ray crystallography

X-ray crystallography offers unparalleled atomic-level resolution of HJ DNA-ligand complexes by crystallising them and reconstructing their 3D structures from X-ray diffraction intensities. Brogden et al. found that a BA-C6 displaces two adenine nucleotides at the HJ’s ACC core, flipping them outward toward the major groove (Fig. 8a) (Brogden et al*. *2007). The acridine units of the ligand formed pseudo-base pairs with thymine bases and participated in π-π stacking with adjacent cytosine and thymine pairs, significantly stabilising the HJ structure. Further stabilisation was provided by spermine molecules binding to the phosphate backbone and a hydrogen bond between the ligand and a cytosine base. These detailed interactions suggest that the C6 bis-acridine locks the HJ into a specific conformation, which could disrupt DNA repair processes by preventing resolution of the junction—a potential strategy for therapeutic intervention in cancer or genetic diseases (Brogden et al. 2007). This level of structural insight is invaluable for rational drug design, enabling precise modifications to enhance ligand efficacy. However, the technique’s labour-intensive nature and the challenge of obtaining high-quality crystals—particularly for flexible structures such as HJs—limits its accessibility. Moreover, it provides only a static snapshot, lacking information on the dynamic aspects of ligand binding. It is the essential primary source for computation and for correlations to spectroscopic methods, and more recently for AI based structure prediction by Alphafold 3.

### Microscale thermophoresis

MST can also be employed to evaluate the binding affinity of ligands with HJ DNA. We recently reported how a bis-acridine dimer, BA-C6 (McGorman et al*. *2023), exhibits cooperative binding and stabilises the stacked-X conformation of HJ DNA with a precise EC_50_ value (4.359 × 10^−7^ M). This indicates a strong and specific interaction, where BA-C6 likely locks the HJ into a compact form, potentially preventing branch migration—a critical step in DNA recombination. In contrast, control experiments with 9-NH_2_-acridine showed no significant binding, showing BA-C6’s selectivity for the HJ structure over other DNA forms (McGorman et al. 2023). Recently, the Hannon group employed MST to investigate the binding properties of a novel metallo-cage compound, Pt-BIMA, with 3WJ and four-way junction (4WJ) DNA (Dettmer et al. 2025). The binding affinity was assessed by observing changes in the thermophoretic movement of the fluorophore-labelled DNA, and the results yielded a *K*_d_ of 1.90 ± 2.27 × 10^−5^ M for Pt-BIMA binding to 3WJ DNA, indicating a moderate affinity. This contrasts with its stronger interaction with 4WJ DNA, where the *K*_d_ was determined to be 8.27 ± 1.11 × 10^−8^ M, reflecting a significantly higher preference for the 4WJ structure (Dettmer et al. 2025). MST’s sensitivity to conformational shifts, combined with its low sample requirements, makes it an excellent choice for comparing compound efficiencies. However, its reliance on fluorescent labelling introduces a risk of altering binding dynamics, and careful optimisation is needed to avoid artefacts such as aggregation, which could skew results.

### Fluorescence-based techniques

Fluorescence techniques have also been employed to examine interactions with HJ DNA. Searcey and co-workers have utilised the intrinsic fluorescence of a 9-aminoacridine derivative to determine the binding affinity of this acridine derivative with HJ DNA. It was reported that 9-aminoacridine showed a strong affinity and selectivity for HJ DNA (*K*_d_ = 0.46 μM) compared with a duplex control (*K*_d_ of 2.44 μM) (Howell et al. 2011). More recent studies have utilised FRET assays to explore ligand-induced conformational changes in HJs. Yin et al. employed a FRET assay to show that VE-822 (Fig. 8c)—a small-molecule ATR kinase inhibitor featuring a pyrazine core with sulphonyl and isoxazole substituents—stabilised HJ formation, evidenced by a dose-dependent decrease in fluorescence (Yin et al. 2021). This suggests that the VE-822 ligand compacts the HJ conformation, quenching the fluorescent signal, which could inhibit its resolution. Additionally, Kepple et al. used 2-aminopurine (2-AP), a fluorescent adenine analogue, to study peptide binding to HJ DNA (Kepple et al. 2008). The fluorescence quenching assay employed showed high-affinity binding with (WRWYCR)_2_ (*K*_d_ = 14 nM) and suggested stacking interactions between peptide aromatic residues and central HJ bases, offering structural insights into the binding mechanism. These fluorescent techniques are rapid and sensitive, making them suitable for high-throughput screening of ligand candidates. However, their dependence on labelling could alter natural interactions, and results may be influenced by environmental factors such as pH or solvent composition, requiring careful experimental design to ensure accuracy.

### Bio-layer interferometry

Bio-layer interferometry (BLI) is a label-free technique (Jug et al. 2024) that measures real-time binding kinetics by detecting shifts in interference patterns as ligands bind to HJ DNA immobilised on a biosensor tip, providing *K*_d_ values. The Hongbo Wang group reported that VE-822 exhibited a *K*_d_ of 8.64 μM for HJ DNA, indicating a robust interaction. This interaction suggests VE-822 could effectively target HJs, potentially disrupting DNA repair pathways in cancer cells, where such processes are often dysregulated (Yin et al. 2021). The kinetic detail offered by BLI—without the need for labelling—provides a dynamic view of ligand binding, making it a valuable tool for understanding interaction mechanisms and optimising therapeutic agents. However, the method requires specialised equipment, which may limit its widespread use, and it has limited detection capabilities for very weak or transient interactions, necessitating higher-affinity compounds for optimal results.

### Thermal melting

Thermal melting of HJs is a key process used to study the stability and structural dynamics of these four-way DNA structures. In a 2016 study, Wang et al. combined UV–vis thermal melting with coarse-grained simulations (3SPN.2 model) to explore the impact of salt concentration on HJ stabilisation (Wang et al. 2016). Salt concentrations up to 200 mM Na^+^ were explored, and the *T*_m_ plateaued at ≥ 200 mM due to strong electrostatic screening stabilising the junction. At 200 mM Na^+^, the simulated *T*_m_ was 5 to 8 K higher than the experimental value, a minor discrepancy of less than 3%, highlighting the 3SPN.2 model’s accuracy. The study also found that higher salt levels favoured the more stable stacked conformations (iso-I and iso-II), critical for HJ function in physiological conditions. Craig et al. also employed thermal melting to investigate the impact of organometallic Au(I) pillarplexes on HJ stability (Craig et al. 2023). They observed that the metallo-pillarplexes stabilise the open form of the HJ, increasing its *T*_m_ by 10 °C for the Ni(II) pillarplex and by 7 °C for the Au(I) derivative.

### Circular dichroism spectroscopy

CD can also be utilised for the analysis of HJs, where it can distinguish between open and stacked-x conformations. Howell et al. employed CD spectroscopy to show that a small acridine-based molecule (Fig. 8b)—synthesised using click chemistry—can induce the formation of stacked-X HJs at room temperature, without high-temperature annealing or divalent metal ions like Mg^2+^ (Howell et al. 2011). The CD spectra displayed enhanced negative signals at 250 nm and positive signals at 277 nm (similar to the effects of the Mg^2+^ control), which when compared to duplex controls (no signal change observed) were found to be specific to the HJ. This CD technique therefore enables analysis of HJ formation/stabilisation in the presence of HJ-binding compounds.

### Optical tweezers

Optical tweezers represent an advanced biophysical tool in molecular biology, facilitating precise manipulation of biomolecules to study their interactions and dynamics (Bustamante et al. 2021). This method employs focused laser beams to generate optical traps that can hold and position microscopic particles, such as DNA molecules tethered to beads, to enable the application of controlled forces and the measurement of molecular responses (Bustamante et al. 2021). Using a directed laser through a microscope objective creates a focused beam, producing a gradient force that secures particles at the focal point. Modifying the beam’s position or intensity enables three-dimensional manoeuvring and the exertion of piconewton-scale forces, making it suitable for investigating biomolecular interactions in a manner that complements traditional molecular techniques (Bustamante et al. 2021; Zaltron et al. 2020). Kaczmarczyk et al. applied this approach to characterise HJs, where they employed Endonuclease I (Endo I)—a junction-resolving enzyme from bacteriophage T7—to reveal insights into the mechanical stability and conformational properties of the junction (Kaczmarczyk et al. 2022). Integrating optical tweezers with ensemble-based methods, such as fluorescence microscopy, can further enhance the analysis of HJ-drug interactions, offering real-time observations of binding events and structural alterations. This combined strategy provides a broader molecular perspective on therapeutic mechanisms, improving our comprehension of how agents modulate DNA repair pathways and supporting the advancement of targeted therapies for conditions like cancer, where HJ dysregulation is implicated.

### Atomic force microscopy

AFM has been pivotal in examining HJs and has been used to describe how DNA supercoiling influences these junctions, favouring a folded, parallel conformation over the antiparallel form seen in synthetic models (Mikheikin et al. 2006). In addition, time-lapse AFM in aqueous environments further enables real-time observation of conformational changes of HJ DNA, offering insights into dynamic processes like DNA replication and repair. The use of force spectroscopy mode in AFM measures mechanical properties by applying force to molecules, unveiling the stability of DNA and protein-DNA interactions, as detailed in Lyubchenko and Shlyakhtenko’s work (Lyubchenko and Shlyakhtenko 2009). In their protocols, force spectroscopy was applied to analyse the dynamics of synaptic SfiI-DNA complexes, where surfaces functionalised with silatranes enabled stable sample immobilisation, allowing measurement of intermolecular forces and revealing insights into complex stability and dissociation kinetics. This approach has broader implications for studying HJ-related dynamics, such as branch migration observed in time-lapse imaging of Holliday junctions on APS-mica surfaces.

### Nuclear magnetic resonance 

NMR has been employed to probe the structural dynamics and metal-ion binding sites of HJs, revealing insights into their conformational preferences and cation interactions in solution (van Buuren et al. 2000). In this work, van Buuren et al. investigated the stacking behaviour and metal-ion localisation with HJ DNA, using cobalt(III) hexammine as a proxy for magnesium ions. If intermolecular NOE contacts are observed between HJ protons and the a(m)mine protons of the cobalt complex in NOESY spectra, it indicates binding at specific sites near the junction. The influence of cobalt(III) hexamine on HJ conformation was assessed by monitoring chemical shift changes and NOE patterns in the presence of the complex compared to magnesium alone, confirming identical A/D stacked conformer preference (>80%) and fast-exchange binding dynamics for both. This enabled the authors to map the binding site of cobalt(III) hexammine precisely, where base-pair residues showed strong NOE interactions, demonstrating high specificity for the negatively charged pocket at the HJ core.

 Table 5 summarises these methods.

**Table 5:** Techniques used to characterise HJ interactions

Technique	Example application
Gel electrophoresis	Visualise binding, conformational changes and cleavage of HJ DNA in the presence of ligands (McGorman et al. 2023; Howell et al. 2011; Craig et al. 2023; Guo et al. 1989)
X-ray crystallography	Provides atomic-level resolution of HJ DNA-ligand complexes by crystallising them and reconstructing their 3D structures from X-ray diffraction intensities(Brogden et al. 2007)
Microscale thermophoresis	Direct measurement of binding affinity of ligands with HJ DNA. Binding to FAM and Cy5-labelled 4WJs has been studied with MST (Dettmer et al. 2025; McGorman et al. 2023)
Fluorescence techniques	The intrinsic fluorescence of acridines, and FRET-based assays have been performed to evaluate binding, stabilisation and compaction of HJ DNA (Kepple et al. 2008; Yin et al. 2021; Howell et al. 2011)
Biolayer interferometry	Measures real-time binding kinetics by detecting shifts in interference patterns as ligands bind to HJ DNA that immobilised on a biosensor tip (Yin et al. 2021)
Thermal melting	Used as a standalone method and in conjunction with computational modelling to investigate thermal stability of HJs (Craig et al. 2023; Wang et al. 2016)
Circular dichroism	Can distinguish between open- and stacked-X HJ, by observing enhanced negative signals at 250 nm and positive at 277 nm (Howell et al. 2011, 2012)
Optical tweezers	Measures force-induced conformational dynamics and enzyme processing of HJ as a biophysical tool (Kaczmarczyk et al. 2022)
AFM	Visualises supercoiling effects on HJ geometry, time-lapse imaging of conformational changes, and force spectroscopy for mechanical stability as a biophysical tool (Mikheikin et al. 2006; Lyubchenko and Shlyakhtenko 2009)
NMR	Allows for the determination of compound contacts with HJ DNA to build an accurate model for compound association/binding (van Buuren et al. 2000)

## Conclusion

Understanding the interactions between drug molecules and nucleic acids is crucial for the development of targeted therapeutics and thereby exploring methods that characterise interactions with canonical and non-canonical DNA is of great importance. This review highlights the critical role of advanced analytical techniques—ranging from biophysical techniques to molecular assays—in elucidating the intricate relationships between these DNA architectures and various molecular agents. These methods have collectively provided a robust framework for understanding how compounds interact with specific DNA sequences and/or structures, revealing their structural dynamics and potential as viable targets for novel treatments.

Despite the sophisticated assays employed for this analysis, significant gaps remain that limit our understanding and the application of these molecular agents in biological systems. Many of the techniques discussed offer a snapshot into the interactions occurring between the drug molecules and their biological target. This can obscure transient binding events or subtle structural changes that are critical for biological function. More recent techniques, such as in-liquid AFM and optical tweezers, can provide real-time analysis of conformational changes and the dynamic properties of the interactions, which offer great promise for the evaluation of interactions of non-canonical nucleic acids. However, a significant challenge that remains is the translation of the insights gained from biophysical assays to the complex environment of a living cell. Many techniques, while highly sophisticated and powerful, are performed under simplified conditions that do not represent the myriad of competing biomolecules or the dynamic cellular process that may influence binding affinity and specify when these compounds are applied in living systems.

The next phase of this research should aim to overcome these limitations through innovative approaches and the integration of multiple technologies within a single application. Future analyses of non-canonical nucleic acids may depend on harmonising assays that preserve sequence context across platforms, while providing technique-specific readouts that deliver complementary insights. This type of approach can ensure a more in-depth understanding of the complex processes underpinning non-canonical nucleic acids and how they can be modulated by small molecules. By combining established biophysical methods with these innovations, including advanced simulations and hybrid assays, targeting specific nucleic acid structures and sequences is poised to deliver innovative solutions to some of the most pressing challenges in modern medicine, particularly in precision oncology and gene regulation.

## Data Availability

No datasets were generated or analysed during the current study.
